# Analysis of Differentially Expressed Genes Related to Resistance in Spinosad- and Neonicotinoid-Resistant *Musca domestica* L. (Diptera: Muscidae) Strains

**DOI:** 10.1371/journal.pone.0170935

**Published:** 2017-01-26

**Authors:** Dorte H. Højland, Michael Kristensen

**Affiliations:** Department of Agroecology, Aarhus University, Slagelse, Denmark; Institute of Zoology Chinese Academy of Sciences, CHINA

## Abstract

**Background:**

The housefly is a global pest that has developed resistance to most insecticides applied against it. Resistance of the spinosad-resistant strain 791spin and the neonicotinoid-resistant 766b strain is believed to be due to metabolism. We investigate differentially expressed genes in these two resistant strains related to metabolism in comparison with an insecticide-susceptible reference strain.

**Results:**

Genes involved in metabolism of xenobiotics were primarily up-regulated in resistant flies with some differences between resistant strains. The *cyp4g98* and *cyp6g4* genes proved interesting in terms of neonicotinoid resistance, while *cyp4d9* was overexpressed in 791spin compared to spinosad-susceptible strains. GSTs, ESTs and UGTs were mostly overexpressed, but not to the same degree as P450s. We present a comprehensive and comparative picture of gene expression in three housefly strains differing significantly in their response to insecticides. High differential expression of P450s and genes coding for cuticle protein indicates a combination of factors involved in metabolic neonicotinoid and spinosad resistance.

**Conclusion:**

Resistance in these strains is apparently not linked to the alteration of a single gene but is composed of several changes including differential expression of genes encoding metabolic detoxification enzymes.

## Introduction

The housefly (*Musca domestica* L.) is a global pest and mechanical vector of more than 100 human and animal pathogens, causing diseases such as salmonellosis, cholera and typhoid fever [1, 2]. While chemical insecticides are still the first line of defense against disease transmission and crop damage by insect pests, their efficacy is being compromised by resistance [3]. Resistance to one or more insecticides has been reported in more than 500 insect and mite species [4].

For centuries, people have been making use of the insecticidal properties of nicotine [5]. Nicotine mimics acetylcholine (ACh) in nerve signaling, causing the development of the insecticide group of neonicotinoids. They are potent selective agonists of insect-nicotinic acetylcholine receptors (nAChRs), causing irreversible blockage of postsynaptic receptors [6, 7], resulting in convulsions and paralysis. Some of the most important members of the group are thiamethoxam, a precursor of clothianidin [8] and imidacloprid, which has good contact action and low human toxicity [9]. Mutations in nAChRs can cause decreased binding affinity of the insecticide to the receptor by desensitization of the receptor or altering the structure [10, 11]. Furthermore, treatment with the synergist piperonyl butoxide (PBO) can indicate resistance caused by increased metabolism by enzymes [12, 13], e.g. due to increased expression of metabolism-associated genes or mutations which alter the effect of the metabolizing enzymes [14–18]. In short, xenobiotics are metabolized in three phases, each with specific metabolic steps. In phase I reactions, cytochrome P450 monooxygenases (P450) primarily oxidate toxic molecules, making them more water-soluble. The process is continued by glutathione-S-transferase (GST) and UDP-glucuronosyltransferase (UGT), which conjugate with the oxidized compounds, producing even more soluble metabolites, which are easier to excrete. Finally, the toxic compounds are transported out of the cell by ABC transporters [18].

Spinosad is another good example of using natural compounds as basis for an insecticide group [19]. Spinosad is a mixture of two macrocyclic lactones: spinosyn A and spinosyn D isolated from the actinomycete bacterium *Saccharopolyspora spinosa* and has been developed as a commercial insecticide [20]. Its mode of action is unique as its primary target site appears to be a subtype of the nAChRs with a proposed secondary target site at the GABA-gated chloride channel [21]. Spinosad causes tremors and involuntary muscle contractions, leading to paralysis [20, 22]. Early work with *Drosophila* has shown nAChRs, especially the α6 subunit, to be important in spinosad resistance [23]. However, alterations of the nAChR α6 subunit seemed not to be involved in resistant houseflies from the US [24] or Denmark [25].

The 766b housefly strain was collected in 2005 and has to our knowledge never been exposed to neonicotinoid insecticides, and the farm has no special record of fly problems or control problems. However, this strain showed resistance to the neonicotinoids thiamethoxam (28-fold) and imidacloprid (140-fold). Resistance ratios were not altered by further selection with either of the two neonicotinoids, and resistance levels did not change as a result of adaption to the laboratory [26]. Treating 766b houseflies with the synergist PBO lowered the resistance ratios, suggesting metabolic resistance. Furthermore, another study showed low expression of a specific acetylcholine receptor subunit in 766b, which could cause decreased binding of neonicotinoids [6, 26].

In 1997, prior to the introduction of spinosad, a survey was conducted to determine baseline susceptibility of spinosad in Danish houseflies. Here a multi-resistant field strain, 791a, was found to be resistant to pyrethroids, spinosad, neonicotinoids and some anticholinesterases [27, 28]. The 791spin strain is a spinosad-selected strain derived from 791a. Selection with spinosad caused diminishment of the resistance towards fipronil, imidacloprid and thiamethoxam seen for the parental 791a strain [25]. Selection with spinosad did not alter the spinosad resistance levels in 791spin compared to the parental 791a strain, maintaining a resistance level of 21-fold and 6-fold for females and males, respectively. The 6-fold resistance is within the natural variation of field strains, classifying males as susceptible and females as resistant. Work regarding the sex-specific resistance [29] hypothesized that the resistance factor of the 791spin strain is located on autosome III, the same position as the male-determining factor. Work on expression of three P450 genes, formerly shown to be of importance in resistance, showed overexpression in resistant strains as well as sex-linked overexpression [25].

Furthermore, additional six P450 genes have been analyzed by qPCR for differences in expression between resistant and susceptible flies [30]. For most of these genes, expression was significantly higher in resistant flies. Four genes, possibly responsible for spinosad resistance, were emphasized with *cyp4g2* as the most likely candidate. However, multiple contributors might be involved. The recent addition of the housefly genome [31] has increased the possibilities in gaining more details with regard to resistance in houseflies and for example transcriptome data can be assessed based on the *Musca* genome rather than the *Drosophila* genome or other resources.

Here, we assess the differential expression pattern of 791spin and 766b in comparison with the insecticide-susceptible reference strain WHO-SRS. We looked into P450s as well as other metabolism-associated enzymes. Furthermore, we compare the two resistant strains as 791spin is spinosad-resistant, but imidacloprid-susceptible and 766b is susceptible to spinosad, but resistant to imidacloprid. By including two resistant strains with different resistance profiles, there is focus on genes overexpressed due to a specific resistance profile and not just genes differentially expressed in former field strains compared to a very susceptible laboratory strain.

## Materials and Methods

### Housefly strains

Housefly breeding followed standard laboratory conditions [32].

The insecticide-susceptible standard reference strain WHO-SRS was received in 1988 from the Department of Animal Biology, University of Pavia, Italy.

The spinosad-selected 791spin strain was established by selection of the spinosad-resistant field strain 791a [25]. The initial selection of 791spin was made by 24 h non-choice feeding sugar impregnated with 71 μg spinosad per g sugar; males (N = 573, 9% survival) and females (N = 406, 32% survival). Selection was repeated in generations 2, 5, 7, 10, 13, 18 and 22 after the initial selection, with increasing concentrations of spinosad. The strain is retained by regular annual selections with spinosad-impregnated sugar. 791spin females were 21-fold spinosad-resistant at LC_50_, whereas 791spin male house flies were 6-fold resistant, which is considered to be within the natural variation in spinosad toxicity of susceptible Danish field populations [33]. 791spin flies were considered susceptible to imidacloprid by having low resistance factors (2-fold) at LC_50_ [25]. The strain is retained by regular selections with spinosad-impregnated sugar with 3.2 mg spinosad per g sugar, for a maximum of 72 hours.

The neonicotinoid-resistant strain 766b is a laboratory strain derived from a field-collected sample of houseflies (68 males and 50 females) collected in the context of a resistance survey in 2005 [34]. Female 766b houseflies were 28- and 140-fold resistant at LC_50_ to thiamethoxam and imidacloprid, respectively. Neither resistance level was altered by selection with the insecticide. However, in the way it was performed, it can’t be excluded that a different selection scheme would have given another result [26]. 766b flies were 5-fold resistant to spinosad, therefore within the range of susceptibility of Danish field houseflies [25].

### Insecticide treatment of houseflies

Five to seven-day-old adult female houseflies were subjected to a non-choice feeding test with spinosad (88%, 76.1% spinosyn A and 11.9% spinosyn D, DOW AgroSciences). The insecticide was diluted with analytic-grade acetone and impregnated on sugar. Females of the spinosad-selected strain 791spin were given 2 mg spinosad per g sugar [25]. Spinosad-susceptible flies were given acetone-coated sugar as a control. All flies had access to water, milk and sugar *ad libitum* before trials. A number of fly batches ranging from 130 to 500 specimens were placed in cages with full access to water and were given excess of granular sugar in a small Petri dish as the only food. The feeding tests were carried out at 25–26°C, 60–65% RH in continuous light. Twenty-four hours upon test start living and fresh looking houseflies were collected by vacuum suction, immediately sedated by cold and killed by freezing. The flies were hereafter kept at -80°C until RNA extraction.

### RNA extraction

Total RNA from whole bodies of houseflies was extracted using the RNeasy Maxi Kit (Qiagen). Pools of flies (approx. 1.2 g equivalent to 60 flies) were thoroughly ground with liquid nitrogen, a mortar and pestle and homogenized with buffer-added β-mercaptoethanol supplied by the RNeasy Kit according to the manufacturer’s protocol. Isolated RNA was DNase-treated and concentrated using the RNeasy MinElute Kit (Qiagen). Gel electrophoresis and spectrophotometry (NanoDrop) was performed to assess the integrity and the concentration of each RNA sample, which was dissolved in RNase-free water and stored at -20°C until use. Three biological replicates of all three strains were obtained.

### Gene expression quantification by RNAseq

For comparison of gene expression nine cDNA libraries was prepared with mRNA selection and fragmentation, strand-specific cDNA synthesis with insert size of 150–400 bp and ligation of adaptors; size selection, PCR amplification, library purification and QC and individual indexing for sequencing. The cDNA libraries were prepared from a) 7 μg RNA from female WHO-SRS, b) 3.7 μg RNA from female WHO-SRS, c) 4.9 μg RNA from female WHO-SRS), d) 7 μg RNA from female 766b, e) 3.1 μg RNA from female 766b, f) 3.6 μg RNA from female 766b, g) 7 μg RNA from female 791spin, h) 4.4 μg RNA from female 791spin, i) 5.6 μg RNA from female 791spin. Some of the samples were also used for qPCR analysis in earlier work.

Sequencing was performed on HiSeq 2500 v3 with rapid run mode (2× 150 bp paired-end reads), and reads were assorted according to indexes. The yield of the nine samples ranged from 2,328 to 2,778 Mb. A total data set of 23,196 Mb was filtered for quality and sorted according to the contig index. Mapping of reads to the housefly genome (GenBank WGS Project: AQPM01) was performed using STAR (version 2.3.0e, http://code.google.com/p/rna-star/). Raw read counts were created using HTSeq (http://www-huber.embl.de/users/anders/HTSeq/doc/overview.html). Only reads with unique mapping positions were considered for read counting. Paired-end reads that were mapped to the same reference with about the expected insert size were counted as one read. Paired-end reads that were mapped to different references or with an unexpected insert size were counted as two reads. If only one read of a pair was mapped, it was counted as one read. Single-end reads were used straightforwardly. Only reads overlapping exon-features were counted. All reads mapping to features with the same identifier were summed. Hereby, the "gene" attribute was used as feature identifier. Reads mapping to multiple features with different identifier were ignored for read counting. A Trimmed Mean of M-values (TMM) normalization was performed using the edgeR (http://bioconductor.org/packages/release/bioc/html/edgeR.html). Prior to differential expression analysis, features with very low expression values were removed as weakly expressed features are in general non-informative. Features had to have a counts-per-million value of more than 1 in at least 3 samples; otherwise they were removed, resulting in the removal of 4,285 of the 14,676 features. All further analysis steps were performed with the remaining 10,391 features. Preparation of cDNA, sequencing, mapping and expression profiling was performed by Eurofins MWG GmbH (Ebersberg, Germany).

### Annotation and functional classification

Annotation of assembled sequences was carried out using BLASTX searches against the NCBI non-redundant protein sequence database in the Blast2GO program. Sequences that shared similarities with known protein sequences in BLASTX searches with significant similarity (E<1e^-10^) were identified using the online tool InterProScan 5.0.

## Results

We obtained close to 102 million RNA-seq reads for the susceptible reference strain WHO-SRS, nearly 98 million for the neonicotinoid-resistant 766b strain and more than 97 million reads for the spinosad-resistant 791spin strain. Reads are made available at NCBI as accessions SAMN06198094-SAMN06198111. More than 90% of the total reads were mapped to the reference database, with nearly all reads mapping uniquely (Table 1). In the differential expression data many genes are represented by more than a single contig. An example of this is the *cyp*12a1 gene, which is represented by two contigs #LOC101890758 and #LOC101898453. The cDNA of *cyp*12a1 is 1,772 bp (GenBank: U86618) and contigs are identical to this. In the 5’ end of the cDNA the initial approx. 530 bp and 150 bp are strongly diverging between the two contigs, whereas the last approx. 1,250 bp are only having 17 single nucleotide differences. This could be an indication of two closely related genes or alternative splicing of one gene. A total of 27,938 sequences (with cutoff *e*-value <1e^-5^) were homologous to proteins in the database. We identified 21,776 sequences (78%), which shared significant similarities (E-value ≤1e^-11^) with known protein sequences and 6,162 sequences (22%), which shared weak similarity with an *e*-value 1e^-4^ to 1e^-10^. Further analysis of BLAST data indicated that just 2.7% of the hits had an *e*-value < 1e^-100^.

**Table 1:** Statistics on RNA-seq yield and read mapping.

Strain	Replicates	Total reads	Mapped reads (%)	Uniquely mapped reads (%)
**WHO-SRS**	A1	35,244,689	92.4	85.5
	A2	34,523,370	94.9	90.6
	A3	32,369,450	94.6	90.3
				
**766b**	B1	34,715,460	93.7	88.3
	B2	30,181,167	93.4	89.4
	B3	32,943,778	94.9	90.1
				
**791spin**	C1	32,605,782	91.6	83.3
	C2	31,162,300	94.5	89.2
	C3	33,315,509	91.5	83.0

### Global expression

The primary data for each of the three housefly strains analyzed in this study can be accessed in S1–S3 Tables. The differential expression analysis of genes in 766b and 791spin compared to the reference strain WHO-SRS showed 10,391 sequences. Of these sequences, 76% did not differ significantly between the 766b strain and the reference strain (*p*-value > 0.05). Furthermore, 68% of the differentially expressed genes (DEGs) were expressed at a higher and 32% of the sequences were expressed at a lower level in 766b compared to WHO-SRS. A similar picture was seen for 791spin, where 73% of the 10,391 genes did not differ significantly from WHO-SRS. Of the remaining 2,810 sequences, 2,020 (72%) sequences were expressed at a higher and 790 (28%) sequences were expressed at a significantly lower level in 791spin compared to WHO-SRS.

A wide range of gene functionalities were represented in the top 20 of highly expressed genes in the resistant strains, including cuticle proteins, P450s and a range of genes with uncharacterized function (S1 and S2 Tables). No clear role of the genes with uncharacterized function was observed, with the exception of the highest expressed feature (LOC101889416). When blasting the sequence of this feature, a gene coding for endothelin-converting enzyme 1 (protease involved in proteolytic processing of pro-peptides involved in vasoconstriction in mammals) was suggested.

The highest differential expression in 791spin was observed for the keratin-associated protein 19 and lysozyme 1, expressed up to 1,122- and 577-fold, respectively. Both of these proteins are important for insects, having a role in the building of structures and in the immune response, respectively. In addition to assessing differential expression between resistant flies and an insecticide-susceptible reference strain, we also looked into differential expression patterns between the two insecticide-resistant strains 791spin and 766b. Of the 10,393 sequences which were observed in comparing these two strains, just 14% were differentially expressed. Most of the DEGs (58%) were expressed higher in 791spin than in 766b.

**Fig 1:**
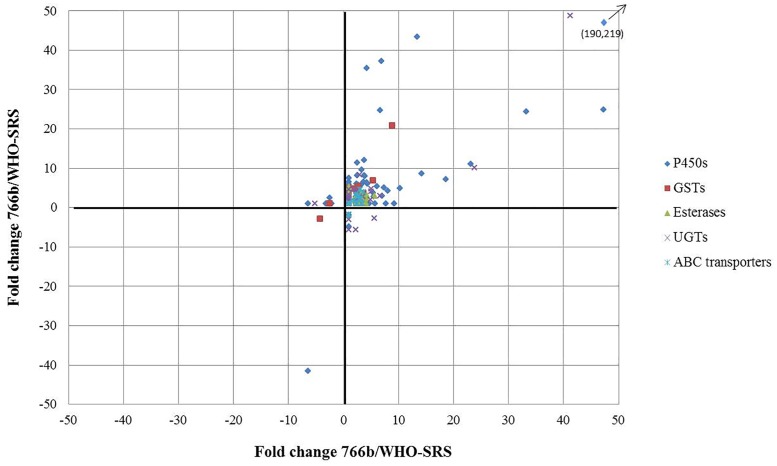
Fold changes of gene expression of P450 genes in resistant strains compared to the reference strain WHO-SRS. Genes in right-upper and left-lower corner are up-regulated and down-regulated in resistant strains, respectively.

The primary focus of the current study is insecticide metabolism-associated genes. Fig 1 shows the gene expression pattern of these genes in 766b and 791spin compared to the reference strain WHO-SRS. Most of the genes related to metabolism were overexpressed in resistant strains; however, not more than 10-fold for most genes. A few genes were overexpressed in one strain and down-regulated in the other strain, while just one P450 (*cyp4g13*) and one GST (*gst1;* contig 895607) were down-regulated in both resistant strains (Fig 1).

### Differential expression of P450 genes

More than 100 contig sequences related to P450 genes were obtained for both resistant strains, with 60–80% being expressed significantly different from the reference strain WHO-SRS. These genes are named based on the naming by Scott *et al*. (2014).

**Fig 2:**
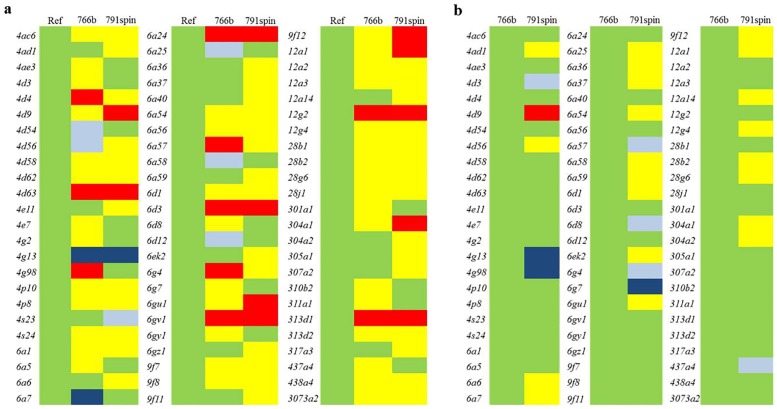
Differential expression of cytochrome P450 genes in housefly strains. A) Differential P450 gene expression between the susceptible reference strain WHO-SRS (Ref) and 766b and 791spin. B) Differential P450 gene expression between the two resistant strains. Genes include those found to be significant differentially expressed in one or both resistant strains. Genes are named according to classification by Scott *et al*. (2014). The full dataset is available in S1–S3 Tables. Colors represent; down-regulation > 5-fold (dark blue), down-regulation < 5-fold (light blue), no fold change (green), up-regulation < 5-fold (yellow), up-regulation > 5-fold (red).

The gene expression pattern of P450 genes differentially expressed in either 766b or 791spin compared to the reference strain, ranging from high down-regulation (dark blue) to high up-regulation (red), is given in Fig 2A. Most of the P450 genes (66%) were not differentially expressed between the two resistant strains (Fig 2B). However, three genes showed large (>8-fold) difference of expression for the two resistant strains; *cyp4g9*, *cyp4g98*, *cyp6g7*.

Based on the pattern in Fig 2A, ten P450 genes of interest were selected along with nine genes tested earlier with quantitative PCR and believed to be of importance in resistance. The exact expression levels and related *p*-values for these 19 P450 genes are given in Table 2. Of the nine genes believed to be important in resistance all but two (*cyp6a36* and *cyp6a37*) were significantly overexpressed in the 766b strain and all, but one (*cyp4g2*) was significantly overexpressed in the 791spin strain (Fig 2A, Table 2). Four of the nine genes did not differ between resistant flies, while one (*cyp6g4*) was overexpressed in 766b and four were overexpressed in 791spin (Fig 2B).

**Table 2:** Differential expression of metabolism-associated genes in housefly strains.

				WHO-SRS vs 766b		WHO-SRS vs 791spin		766b vs 791spin	
Gene group	Gene name	N	#Feature (LOC101)	*p*-value^1^	Fold change^2^	*p*-value^1^	Fold-change^2^	*p*-value^1^	Fold-change^2^
**Cytochrome P450**	*cyp4d9*	1	897209	0.0021	4.3	0.0000	36	0.0000	8.3
	*cyp4d63*	1	894425	0.0000	190	0.0000	218	0.6190	
	*cyp4d2*	1	887882	0.0082	3.5	0.3364		0.0823	
	*cyp4g13*	1	887550	0.0062	-6.5	0.0000	-42	0.0068	-6.4
	*cyp4g98*	1	889105	0.0000	9.2	0.7893		0.0001	-8.1
	*cyp6a1*	1	889365	0.0229	3.2	0.0007	5.6	0.2264	
	*cyp6a24*	1	892246	0.0000	47	0.0000	25	0.0905	
	*cyp6a36*	1	889539	0.4812		0.0007	3.8	0.0060	2.9
	*cyp6a37*	2	890199	0.4827		0.0119	2.3	0.0667	2.1
			890373	0.9517		0.0117	2.1	0.0099	
	*cyp6d1*	2	899746	0.0003	4.2	0.0000	6.4	0.2725	2.2
			900791	0.0000	3.8	0.0000	8.2	0.0150	
	*cyp6d3*	1	899585	0.0000	23	0.0000	11	0.0544	
	*cyp6g4*	1	898562	0.0000	10	0.0000	5.0	0.0419	-2.1
	*cyp6g7*	1	900444	0.0067	5.7	0.5473		0.0010	-9.8
	*cyp6g1*	1	894510	0.0001	14	0.0012	8.8	0.4064	
	*cyp9f12*	1	900658	0.0013	3.8	0.0000	12	0.0046	3.2
	*cyp12a1*	2	898453	0.0001	6.9	0.0000	37	0.0003	5.4
			890758	0.0009	6.7	0.0000	25	0.0123	3.7
	*cyp12a2*	1	889857	0.0000	5.4	0.0010	3.8	0.3667	
	*cyp12g2*	1	893522	0.0000	33	0.0000	24	0.2296	
	*cyp313d1*	1	890728	0.0014	13	0.0000	43	0.0828	
**Glutathione-S-transferase**	*gst1*	14	897621	0.0006	3.2	0.0000	62	0.0000	19
			895036	0.0000	8.8	0.0000	21	0.0061	2.4
			895607	0.0000	-4.2	0.0000	-2.9	0.1561	
	*gst2*	2	897094	0.0001	3.3	0.0041	2.3	0.2465	
			897277	0.0365	1.9	0.0000	4.9	0.0018	2.5
	*gstD7*	1	897797	0.7188		0.0221	3.4	0.0504	
	*gst- theta-1*	5	897781	0.0516		0.0391	1.9	0.9041	
			888110	0.0278	-2.5	0.2343		0.0010	4.1
									
**Esterases**	*Esterase B1*	14	896625	0.0004	4.2	0.0042	3.2	0.4730	
			898347	0.0021	3.4	0.0448	2.2	0.2599	
			900490	0.0027	3.2	0.0004	4.0	0.5570	
	*Esterase FE4*	1	890018	0.5695		0.0050	3.7	0.0234	2.8
	*Esterase 5A*	1	889364	0.0000	4.3	0.0266	2.1	0.0394	-2.0
	*Acetylcholinesterase*	1	890429	0.2075		0.0002	5.2	0.0120	3.0
									
**ATP-binding cassette transporter**	*ABC transporter B6*	1	899501	0.4154		0.1364		0.0217	-1.5
	*ABC transporter G1*	7	894746	0.0059	3.6	0.0036	3.9	0.8637	
			897386	0.0275	2.6	0.0149	2.9	0.8076	
			894584	0.0028	3.1	0.0234	2.4	0.4505	
	*ABC transporter G4*	5	890630	0.0169	3.0	0.0012	4.5	0.3612	
			890462	0.0040	3.0	0.0303	2.2	0.4574	
			887291	0.0153	1.8	0.0240	1.7	0.8672	
	*ABC transporter G20*	3	895448	0.0363	2.7	0.0322	2.7	0.9565	
			889472	0.0278	2.3	0.0016	3.3	0.3149	
			898054	0.0035	2.5	0.0525		0.3148	
	*ABC transporter G22*	1	890903	0.0793		0.0371	-1.8	0.7376	
									
**UDP-glucuronosyltransferase**	*ugt2a2*	1	888811	0.8406		0.0038	3.3	0.0069	3.0
	*ugt2a3*	3	893116	0.0000	24	0.0000	10	0.0149	-2.4
			893291	0.0403	1.9	0.0000	4.5	0.0075	2.4
			889193	0.0000	5.0	0.0001	4.7	0.8671	
	*ugt2b1*	2	893458	0.0724		0.0033	4.6	0.0000	12
	*ugt2b4*	1	890707	0.0023		0.5358		0.0173	3.8
	*ugt2b7*	1	889773	0.0000	5.1	0.0001	4.0	0.5203	
	*ugt2b13*	3	889322	0.0006	3.0	0.0000	8.4	0.0009	2.9
			890271	0.0052	2.5	0.0000	4.5	0.0822	
			900184	0.3843		0.0033	2.3	0.0362	1.8
	*ugt2b15*	2	895816	0.0174	2.3	0.0001	4.1	0.1111	
			897252	0.4886		0.0121	2.7	0.0697	
	*ugt2b17*	2	899999	0.0689		0.0354	1.7	0.7726	
			899504	0.4665		0.2658		0.6999	
	*ugt2b20*	2	890612	0.1477		0.2247		0.8207	
			890444	0.6040		0.0394	2.1	0.1209	
	*ugt2b31*	1	892660	0.0000	4.9	0.0192	2.0	0.0035	-2.4
	*ugt*	6	889147	0.0000	41	0.0000	49	0.6762	
			892938	0.0141	5.6	0.0141	-2.7	0.0000	-15
			899032	0.0000	2.3	0.0000	-5.7	0.0000	-13

When more than three features were present, representatives are shown. P450 genes are named according to classification by Scott *et al*. (2014). The full dataset is available in S1–S3 Tables.

^1^Fold changes were considered significant if p < 0.05.

^2^Fold change values above zero represent overexpression, while values below zero represent down-regulation in latter strain.

The highest expressed P450 gene was the *cyp4d63*, which was 218-fold and 190-fold overexpressed in 791spin and 766b, respectively. Likewise, *cyp6a24* proved highly expressed in both resistant strains (Table 2). The *cyp313d1* gene represented the second highest expressed P450 gene in 791spin at 43-fold overexpression. This gene is involved in sex determination of houseflies [35] and was also found to be overexpressed (13-fold) in 766b. A few P450 genes were significantly down-regulated in resistant flies, including the *cyp4g13* gene. It was down-regulated 6.5- and 42-fold in 766b and 791spin, respectively (Table 2).

### Differential expression of glutathione-S-transferase genes

Twenty-three sequences related to five GST gene families were observed in the present study (Fig 1, Table 2). Most of the sequences for *gst1* and *gst2* were significantly overexpressed in resistant flies, ranging from 1.9- to 8.8-fold overexpression in 766b and 2.3- to 62-fold in 791spin compared to WHO-SRS, but one *gst1* contig (895607) was down-regulated in both resistant strains (Table 2).

### Differential expression of esterase genes

Three esterase genes and one acetylcholinesterase gene were observed in the dataset, all of which were overexpressed in 791spin, but esterase FE4 and acetylcholinesterase were not overexpressed in 766b (Fig 1, Table 2).

### Differential expression of ABC transporter genes

Seven families of ABC transporters were observed in the dataset, but DEGs between resistant and susceptible flies were only observed for the G family (Table 2). Significantly expressed sequences were all expressed more than 1.7-fold in the resistant strains compared to the reference strain, with the exception of the ABC transporter G22 gene, which was down-regulated in 791spin compared to WHO-SRS, but not different in 766b. No differential expression pattern was observed between the two resistant housefly strains (Table 2).

### Differential expression of UDP-glucuronosyltransferase genes

In the current study, 14 genes were obtained for UDP-glucuronosyltransferase, primarily of the *ugt2b* subfamily (Table 2). Six genes (*ugt2a3*, *ugt2b7*, *ugt2b13*, *ugt2b31*, *ugt2b33* and *ugt2c1*) were more than 1.9-fold overexpressed in both strains of resistant flies in addition to a single sequence for the general UGT gene, which were more than 40-fold overexpressed in resistant flies. Most of the UGT genes were not different between resistant flies. However, a few contigs were differentially expressed.

## Discussion

The high level of uniquely mapped sequences (Table 1) suggests a solid dataset for analysis of differential expression between resistant and susceptible houseflies, which was further ensured by the use of three biological replicates of each strain. Approximately one of four transcripts was a DEG in the resistant strains compared to the susceptible strain. This difference is attributed to strain specific differences originating from different geographic origin, adaptation to laboratory breeding, and in some case the insecticide resistance phenotype. This suggests that the strains are rather similar globally, which correlates with all three strains having adapted to laboratory life over several years. Laboratory adaption could have minimized the differences related to field performance. This would mean that differences that are related to maintenance of resistance or other metabolism processes are still differentially expressed.

In the spinosad-resistant 791spin strain, several genes important for survival of houseflies were found to be highly differentially expressed when they were compared to the susceptible reference strain, including keratin-associated protein 19 and lysozyme 1. Keratins and keratin-associated proteins are important in making strong chitin structures when constructing the cuticle. Lysozymes are part of the innate immune system, where it is capable of damaging the bacterial cell wall, which is protecting the insect against infections. Both of these genes were overexpressed more than 500-fold in spinosad-resistant houseflies. Keratin-associated protein 19 was also overexpressed in 766b flies but not to the same extent as in 791spin. A highly overexpressed DEG in the current study was a gene coding for endothelin-converting enzyme 1. This gene was overexpressed more than 400-fold in resistant houseflies, but not differently between the two resistant strains. This enzyme is involved in the proteolytic processing of endothelins, which in mammals are vasoconstricting peptides having a key role in vascular homeostasis. Many genes coding for cuticle proteins in the three life stages of the housefly were found to be significantly overexpressed. The high level of genes related to the cuticle, such as adult cuticle protein and keratin-associated protein 19 observed in this dataset, suggests the cuticle in resistant flies is more resilient than that found in susceptible flies.

Both resistant strains have been in a laboratory setting for more than ten years, while maintaining the initial resistance levels. The RNA used for the present experiments are the same used earlier [30] and subsequently stored at -80°C. Here, the real-time qPCR experiments were confirmed by the present RNAseq data, and trends observed by RNAseq were equal to qPCR trends showed earlier. Overall, gene expression of the two resistant strains is similar, only 15% were DEGs. Likewise, most of the genes associated with metabolism were similar in the two resistant strains. However, some differences were observed when comparing the two strains, especially for the P450 genes. Cytochrome P450 enzymes are present in large numbers and have a wide substrate spectrum, causing them to be ideal candidates for metabolic insecticide resistance whether due to a single P450 or multiple P450s causing resistance when combined [36]. The 766b strain is resistant to neonicotinoids, but susceptible to spinosad, while 791spin is the opposite; raising potential for identification of specific P450 genes related to either of the resistance profiles. Genes, which are highly expressed in 766b but not so in 791spin, might be important in neonicotinoid resistance and vice versa for spinosad resistance. In order for a gene to be a candidate for resistance, it must be higher expressed in the resistant strain compared to the two susceptible strains. However, a spinosad resistance candidate gene might be slightly higher expressed in 766b than in WHO-SRS despite both strains being susceptible to spinosad. This is due to WHO-SRS being very susceptible due to more than 40 years in laboratory breeding without selection. The same is the case for neonicotinoid resistance genes being slightly overexpressed in 791spin compared to WHO-SRS. By assessing the pattern in Fig 2, possible P450 candidates responsible for neonicotinoid and spinosad resistance can be identified. The overexpressed P450 genes observed in Fig 2 are from diverse P450 families, indicating a complex involvement of multiple P450s in neonicotinoid and spinosad resistance.

In 766b, some genes shown earlier to be of importance in resistance as well as some additional P450 genes were overexpressed when compared to the reference strain WHO-SRS and the imidacloprid-susceptible 791spin strain. These include *cyp4g98* and *cyp6g4*. The *cyp4g98* gene was 9.2-fold and 8.1-fold overexpressed in 766b compared to WHO-SRS and 791spin, respectively. This indicates a role of *cyp4g98* in neonicotinoid resistance. A similar pattern was seen for the spinosad-resistant 791spin strain, where *cyp4d9* was overexpressed compared to spinosad-susceptible strains. In fact, the *cyp4d9* gene was overexpressed in both resistant strains, but less that 5-fold in 766b and 36-fold in 791spin and more than 8-fold compared to WHO-SRS and 766b, respectively. This gene might be the next step in understanding spinosad resistance.

More than half of the P450 genes tested here were not different between the two resistant strains, indicating that the two strains are similar in many ways in terms of genetic background as well as the WHO-SRS proving a very susceptible reference strain.

Earlier work done on the three housefly strains described here showed differential gene expression for a range of P450 genes. These include *cyp4g2*, *cyp6a1*, *cyp6d1*, *cyp6d3* and *cyp6g4* [26, 30, 37]. In the present study the same RNA samples were used enabling us to compare qPCR and transcriptome data. Here, we also found all the *cyp6* genes to be overexpressed in resistant flies, while *cyp4g2* did not differ significantly from WHO-SRS in 791spin.

The CYP4G1 enzyme from *Drosophila* plays a key role in restriction of water loss during development, ultimately protecting insects against dehydration [38, 39]. The housefly ortholog *cyp4g2* is inducible by permethrin in the resistant ALHF strain, indicating a minor role for *cyp4g2* in permethrin resistance of this strain [40]. The *cyp4g2* gene is located on chromosome III, just like the male-determining factor of 791spin, and has been found to be overexpressed in resistant flies [29, 30]. Since *cyp4g2* is not overexpressed in 791spin in the current study, it seems not be involved in 791spin resistance despite it being the best P450 candidate tested earlier. From the same family, the *cyp4d63* gene was almost 220-fold and 190-fold overexpressed in 791spin and 766b flies compared to the reference strain, being the highest P450 DEG. The *cyp4g63* gene is the housefly ortholog to the *cyp4d8* gene from *Drosophila melanogaster* [31] and was found to be increased upon selection with the toxin α-amanitin, which is produced by poisonous mushroom species [41, 42]. The high expression level in resistant flies of *cyp4d63* raises the potential of this gene as a general metabolism gene. Furthermore, other genes were found to be highly expressed in both 791spin and 766b flies, including *cyp6a24*, *cyp12g2* and *cyp313d1*, all of which were expressed more than 13-fold in resistant flies, but not differentially expressed between resistant strains. More work focusing on highly expressed genes obtained in the present study might get us closer to find major players in general resistance against insect neurotoxins in houseflies.

The *cyp6a1* gene was the first insect P450 to be isolated [43] and was overexpressed in the resistant Rutgers and LPR strains [25, 44] as well as in resistant Danish housefly strains, including 766b, 791a and 791spin [25, 26, 30, 37]. The slight overexpression of *cyp6a1* in both resistant strains of the current study and the lack of difference between the two strains suggest that *cyp6a1* is not of importance in spinosad or neonicotinoid resistance.

The *cyp6d1* gene is overexpressed in flies resistant to pyrethroids and neonicotinoids [26, 45–47]. The *cyp6d1* gene appears to be involved in male neonicotinoid resistance in 766b, while female resistance seems to be linked to overexpression of the related *cyp6d3* gene [26]. An overexpression of *cyp6d1* and *cyp6d3* has been observed earlier in spinosad-treated 791spin flies compared to WHO-SRS flies [25, 30]. In the current study, both genes were overexpressed in resistant flies, which correlate with the former studies.

The *cyp6g4* gene is a potential housefly ortholog of *Drosophila cyp6g1* gene (60.7% identity), which has been associated with neonicotinoid resistance [48, 49], and *cyp6g4* is hypothesized to have a similar function. This gene was overexpressed in pyrethroid-resistant populations and was hypothesized to be contributing to pyrethroid resistance of adult Chinese houseflies [50]. The *cyp6g4* gene was overexpressed more than 10-fold in 766b and 5-fold in 791spin compared to the reference strain, in both earlier and current studies, with a significant overexpression in 766b. Clear overexpression of *cyp6g4* in 766b has now been observed by two methods, both suggesting a role in neonicotinoid resistance.

Most of the P450 genes were up-regulated in resistant flies, but a few genes were significantly down-regulated compared to the reference strain. The *cyp4g13* gene was down-regulated 41-fold in 791spin and 6.5-fold in 766b in comparison with WHO-SRS. This gene was also included in a study of laboratory adaption in a newly-collected field strain and was not altered much over time [51]. The present and former studies suggest that *cyp4g13* and other P450 genes not significantly expressed in resistant strains have a more general role in houseflies, not related to resistance. The observed picture for expression of P450 genes is quite complex, which was emphasized in Fig 1 where multiple genes were highly overexpressed and not just a single P450 gene could be named responsible, unlike that seen in several insect species, such as pyrethroid resistance in pollen beetles or DDT resistance in *Drosophila* [52, 53].

GSTs are important detoxification enzymes which can cause resistance to insecticides [54] and provide protection against oxidative stress [55]. A high level of GST activity has been found in 791a, the parental strain of 791spin, along with other laboratory strains and some field strains [56], indicating an effect in resistance. Most of the GST genes in the current study were overexpressed in resistant flies, with the highest level in 791spin. The overexpression of GST genes in resistant flies could suggest a role of GSTs in resistance, especially for *gst1* in spinosad resistance. However, concerning *gst1* we had an interesting observation; the expression of one contig is down-regulated, whereas other *gst1* contigs has increased their expression. This could be explained by changes in differential splicing or more detailed regulation of expression within the gene family.

The family of esterase enzymes can hydrolase ester bonds, which are present in a wide range of insecticides, including organophosphates, pyrethroids and spinosad, but not imidacloprid [15]. Work done on Chinese houseflies has shown importance of esterases for spinosad resistance [57]. In the housefly, esterases are also affected by PBO, which could cause some of the synergist effect observed in earlier work on 791spin [25]. Here, we found four esterase genes all significantly overexpressed in 791spin. The esterase B1 and esterase 5A genes were slightly overexpressed in 766b. Esterase FE4 might have a slight impact on spinosad resistance, but based on the data obtained here esterases are not of major importance for spinosad and neonicotinoid resistance.

Insect UGTs have been shown to play a role in both inactivation and excretion of compounds. Furthermore, UGTs might also play a role in cuticle formation [58] and resistance in the tobacco budworm [59]. Here, overexpression of several UGT genes were observed in the two resistant strains, up to 49-fold for the general UGT gene in 791spin, indicating that UGTs could have a role in unspecific insecticide resistance. However, other sequences related to the same gene were down-regulated for 791spin, so the expression pattern is quite diverse, making sound conclusions difficult.

In recent years, importance of ABC transporters in resistance have been suggested [18]. Eukaryotic ABC transports are related to efflux of toxic compounds, while bacterial ABC transporters also can function in influx of compounds [18]. Furthermore, most of the ABC transporters associated with the efflux of pesticides belong to the subfamilies ABCB and ABCG for the malaria vector *Anopheles stephensi* [60]. Those are the two families which are represented in our data set. However, only moderate overexpression of ABC transporters in resistant strains was observed here, indicating that ABC transporters are of minor importance in spinosad and neonicotinoid resistance in Danish houseflies.

In addition to metabolism associated genes, gene expression of other genes were also analyzed, such as genes related to receptors and stress. The primary site of action for both spinosad and neonicotinoid insecticides is different subsets of the nACh receptor. No general trend of overexpression was observed for nACh receptor subunit genes for either 791spin or 766b. A small effect of spinosyn A has been observed for the voltage-gated calcium channel earlier [61] and in the current study we did observe single sequences coding for the *vgccα2δ3* and *vgccβ4* subunits, which were 5.4-fold and 4.5-fold overexpressed in 791spin flies, respectively. However, in general just small changes in receptor and ion channel genes were observed between the three strains of the current study, indicating little importance of these genes in describing the resistance mechanisms of 791spin and 766b.

Højland *et al*. (2014a) tested changes in expression of multiple genes, which occur when a field strain is adapting to a laboratory setting. Among those genes were the enzyme superoxide dismutase (SOD), which is important for the antioxidant defense and linked to the xenobiotic response [62] and the antibacterial peptide attacin which is part of the non-specific insect immune system [63]. Both of these genes had a drastic decrease in gene expression over time (approximately two years), representing little use of either of these genes in a laboratory setting [51]. All three strains mentioned in the present study have adapted to the laboratory for more than ten years, so it is not surprising that differences in gene expression of defense genes between these three strains were limited.

Several experiments are upcoming in order to unravel the complex, gene expression-driven, metabolic insecticide resistance mechanisms; i) based on the recently published housefly genome [31] common gene regulatory motifs based on the promoter regions of housefly detoxification genes should be elucidated and have been initiated on a small scale [64], ii) metabolomics studies of insecticide detoxification pathways, iii) functional studies of the catalytic activities of individual housefly detoxification enzymes are warranted, as well as iv) RNAi and/or transgene manipulation of detoxification genes, but the latter two experimental methods are not well established in the housefly.

## Conclusions

The data we observe are strain specific expression patterns originating from the field history of the strains, their resistance profiles as well as their adaptation to laboratory breeding. We found a high expression of certain P450 genes believed to be of interest. Most of the P450 genes were not differentially expressed between resistant strains, but a few showed strain-specific expression patterns. For 766b, those genes were *cyp4g98* and *cyp6g4* and for 791spin, *cyp4d9* was overexpressed compared to spinosad-susceptible strains. These genes might be the next step in understanding imidacloprid and spinosad resistance in houseflies.

GST genes were overexpressed in both resistant strains, but with a possibly more dominant role in 791spin. Members of the ABC transporter G subfamily seem to have small roles in general resistance as these were overexpressed in resistant strains. The importance of UGTs in insecticide resistance is less clear based on our data. The work supports previous work by the authors showing an up-regulation of several metabolism-associated genes in combination with a robust cuticle instead of upregulation of a single gene being the sole contributor to resistance. However, the exact mechanism behind the biochemistry of the observed spinosad and neonicotinoid resistance as well as the molecular mechanism creating the changed up-regulated detoxification system causing a complex resistance profile still remains to be elucidated.

## Supporting Information

S1 Table Raw data for global gene expression and expression of genes related to metabolism in the spinosad-resistant 791spin strain compared to the susceptible reference strain WHO-SRS. Fold change (log2 values), logCPM, P-Value and FDR are provided.
(PDF)

S2 Table Raw data for global gene expression and expression of genes related to metabolism in the neonicotinoid-resistant 766b strain compared to the susceptible reference strain WHO-SRS. Fold change (log2 values), logCPM, P-Value and FDR are provided.
(PDF)

S3 Table Raw data for global gene expression and expression of genes related to metabolism in the spinosad-resistant 791spin strain compared to the neonicotinoid-resistant 766b strain. Fold change (log2 values), logCPM, P-Value and FDR are provided.
(PDF)

## References

[1] Keiding J. The House-fly—biology and control Vector control series: The housefly: World Health Organization; 1986.

[2] Scott HG, Littig KS. Flies of public health importance and their control Flies of public health importance and their control: US department of Health, Education and Welfare; 1962.

[3] David JP, Ismail HM, Chandor-Proust A, Paine MJI. Role of cytochrome P450s in insecticide resistance: impact on the control of mosquito-borne diseases and use of insecticides on Earth. Philosophical Transactions of the Royal Society B-Biological Sciences. 2013;368(1612). 10.1098/rstb.2012.0429 PMC3538419 23297352

[4] Georghiou G, P. Overview of Insecticide Resistance. Managing Resistance to Agrochemicals. ACS Symposium Series. 421: American Chemical Society; 1990. p. 18–41.

[5] Steppuhn A, Gase K, Krock B, Halitschke R, Baldwin IT. Nicotine's defensive function in nature. PLoS biology. 2004;2(8):E217 10.1371/journal.pbio.0020217 15314646 PMC509292

[6] Markussen MDK, Kristensen M. Low expression of nicotinic acetylcholine receptor subunit Md alpha 2 in neonicotinoid-resistant strains of Musca domestica L. Pest Management Science. 2010;66(11):1257–62. 10.1002/ps.2007 20730783

[7] Ware GW, Whitacre DM. The pesticide book: MeisterPro Information Resources; 2004.

[8] Nauen R, Ebbinghaus-Kintscher U, Salgado VL, Kaussmann M. Thiamethoxam is a neonicotinoid precursor converted to clothianidin in insects and plants. Pesticide Biochemistry and Physiology. 2003;76(2):55–69.

[9] Chao SL, Casida JE. Interaction of imidacloprid metabolites and analogs with the nicotinic acetylcholine receptor of mouse brain in relation to toxicity. Pesticide Biochemistry and Physiology. 1997;58(1):77–88.

[10] Bass C, Puinean AM, Zimmer CT, Denholm I, Field LM, Foster SP, et al The evolution of insecticide resistance in the peach potato aphid, Myzus persicae. Insect Biochem Mol Biol. 2014;51:41–51. 10.1016/j.ibmb.2014.05.003 24855024

[11] Kavi LAK, Kaufman PE, Scott JG. Genetics and mechanisms of imidacloprid resistance in house flies. Pestic Biochem Phys. 2014;109:64–9. 10.1016/j.pestbp.2014.01.006 24581385

[12] ffrench-Constant RH, Daborn PJ, Le Goff G. The genetics and genomics of insecticide resistance. Trends in Genetics. 2004;20(3):163–70. 10.1016/j.tig.2004.01.003 15036810

[13] Li X, Schuler MA, Berenbaum MR. Molecular mechanisms of metabolic resistance to synthetic and natural xenobiotics. Annual review of entomology. 2007;52:231–53. 10.1146/annurev.ento.51.110104.151104 16925478

[14] Scott JG. Cytochromes P450 and insecticide resistance. Insect biochemistry and molecular biology. 1999;29(9):757–77. 10510498 10.1016/s0965-1748(99)00038-7

[15] Montella IR, Schama R, Valle D. The classification of esterases: an important gene family involved in insecticide resistance—a review. Memorias do Instituto Oswaldo Cruz. 2012;107(4):437–49. 22666852 10.1590/s0074-02762012000400001

[16] Scott JG, Liu N, Wen Z. Insect cytochromes P450: diversity, insecticide resistance and tolerance to plant toxins. Comparative biochemistry and physiology Part C, Pharmacology, toxicology & endocrinology. 1998;121(1–3):147–55. 10.1016/s0742-8413(98)10035-x 9972456

[17] Enayati AA, Ranson H, Hemingway J. Insect glutathione transferases and insecticide resistance. Insect Molecular Biology. 2005;14(1):3–8. 10.1111/j.1365-2583.2004.00529.x 15663770

[18] Dermauw W, Van Leeuwen T. The ABC gene family in arthropods: Comparative genomics and role in insecticide transport and resistance. Insect Biochemistry and Molecular Biology. 2014;45:89–110. 10.1016/j.ibmb.2013.11.001 24291285

[19] Kirst HA. The spinosyn family of insecticides: realizing the potential of natural products research. Journal of Antibiotics. 2010;63(3):101–11. 10.1038/ja.2010.5 20150928

[20] Sparks TC, Crouse GD, Durst G. Natural products as insecticides: the biology, biochemistry, and quantitative structure-activity relationships of spinosyns and spinosoids. Pest Management Science. 2001;57(10):896–905. 10.1002/ps.358 11695182

[21] Watson GB. Actions of insecticidal spinosyns on gamma-aminobutyric acid responses from small-diameter cockroach neurons. Pesticide Biochemistry and Physiology. 2001;71(1):20–8.

[22] Salgado VL. Studies on the mode of action of spinosad: Insect symptoms and physiological correlates. Pesticide Biochemistry and Physiology. 1998;60(2):91–102.

[23] Perry T, McKenzie JA, Batterham P. A Dα6 knockout strain of< i> Drosophila melanogaster confers a high level of resistance to spinosad. Insect biochemistry and molecular biology. 2007;37(2):184–8. 10.1016/j.ibmb.2006.11.009 17244547

[24] Scott JG. Unraveling the mystery of spinosad resistance in insects. Journal of Pesticide Science. 2008;33(3):221–7.

[25] Markussen MDK, Kristensen M. Spinosad resistance in female Musca domestica L. from a field-derived population. Pest Management Science. 2012;68(1):75–82. 10.1002/ps.2223 21681919

[26] Markussen MDK, Kristensen M. Cytochrome P450 monooxygenase-mediated neonicotinoid resistance in the house fly Musca domestica L. Pesticide Biochemistry and Physiology. 2010;98(1):50–8.

[27] Kristensen M, Jespersen JB, Knorr M. Cross-resistance potential of fipronil in Musca domestica. Pest Management Science. 2004;60(9):894–900. 10.1002/ps.883 15382504

[28] Kristensen M, Spencer AG, Jespersen JB. The status and development of insecticide resistance in Danish populations of the housefly Musca domestica L. Pest Management Science. 2001;57(1):82–9. 10.1002/1526-4998(200101)57:1<82::AID-PS251>3.0.CO;2-8 11455636

[29] Højland DH, Scott JG, Vagn Jensen K-M, Kristensen M. Autosomal male determination in a spinosad-resistant housefly strain from Denmark. Pest Management Science. 2014;70(7):1114–7. 10.1002/ps.3655 24105942

[30] Højland DH, Jensen K-MV, Kristensen M. Expression of Xenobiotic Metabolizing Cytochrome P450 Genes in a Spinosad-Resistant *Musca domestica* L. Strain. PLoS ONE. 2014;9(8):e103689 10.1371/journal.pone.0103689 25165825 PMC4148238

[31] Scott JG, Warren WC, Beukeboom LW, Bopp D, Clark AG, Giers SD, et al Genome of the house fly, Musca domestica L., a global vector of diseases with adaptations to a septic environment. Genome Biol. 2014;15(10):466 10.1186/s13059-014-0466-3 25315136 PMC4195910

[32] Kristensen M, Huang J, Qiao CL, Jespersen JB. Variation of Musca domestica L. acetylicholinesterase in Danish housefly populations. Pest Management Science. 2006;62(8):738–45. 10.1002/ps.1231 16718740

[33] Kristensen M, Jespersen JB. Susceptibility of spinosad in Musca domestica (Diptera: Muscidae) field populations. Journal of Economic Entomology. 2004;97(3):1042–8. 15279289 10.1093/jee/97.3.1042

[34] Kristensen M, Jespersen JB. Susceptibility to thiamethoxam of Musca domestica from Danish livestock farms. Pest Management Science. 2008;64(2):126–32. 10.1002/ps.1481 17972302

[35] Meisel RP, Scott JG, Clark AG. Transcriptome Differences between Alternative Sex Determining Genotypes in the House Fly, Musca domestica. 2015 2015-01-01 00:00:00. 10.1093/gbe/evv128 PMC4524491 26142430

[36] Tao XY, Xue XY, Huang YP, Chen XY, Mao YB. Gossypol-enhanced P450 gene pool contributes to cotton bollworm tolerance to a pyrethroid insecticide. Mol Ecol. 2012;21(17):4371–85. 10.1111/j.1365-294X.2012.05548.x 22515600

[37] Højland DH, Vagn Jensen K-M, Kristensen M. A comparative study of P450 gene expression in field and laboratory Musca domestica L. strains. Pest Management Science. 2014;70(8):1237–42. 10.1002/ps.3681 24227651

[38] Nelson DR, Goldstone JV, Stegeman JJ. The cytochrome P450 genesis locus: the origin and evolution of animal cytochrome P450s. Philosophical Transactions of the Royal Society B-Biological Sciences. 2013;368(1612). 10.1098/rstb.2012.0474 PMC3538424 23297357

[39] Gullan PJ, Cranston P. The Insects: An Outline of Entomology: Wiley; 2005.

[40] Zhu F, Li T, Zhang L, Liu N. Co-up-regulation of three P450 genes in response to permethrin exposure in permethrin resistant house flies, Musca domestica. BMC Physiology. 2008;8:18 10.1186/1472-6793-8-18 18817570 PMC2567968

[41] Mitchell CL, Saul MC, Lei L, Wei H, Werner T. The Mechanisms Underlying α-Amanitin Resistance in *Drosophila melanogaster*: A Microarray Analysis. PLoS ONE. 2014;9(4):e93489 10.1371/journal.pone.0093489 24695618 PMC3973583

[42] Coelho A, Fraichard S, Le Goff G, Faure P, Artur Y, Ferveur J-F, et al Cytochrome P450-Dependent Metabolism of Caffeine in Drosophila melanogaster. PLoS ONE. 2015;10(2):e0117328 10.1371/journal.pone.0117328 25671424 PMC4324904

[43] Feyereisen R, Koener JF, Farnsworth DE, Nebert DW. Isolation and Sequence of Cdna-Encoding a Cytochrome-P-450 from an Insecticide-Resistant Strain of the House-Fly, Musca-Domestica. Proceedings of the National Academy of Sciences of the United States of America. 1989;86(5):1465–9. 2922393 10.1073/pnas.86.5.1465 PMC286717

[44] Cariño F, Koener JF, Plapp FW, Feyereisen R. Expression of the Cytochrome P450 Gene CYP6A1 in the Housefly, Musca domestica. Molecular Mechanisms of Insecticide Resistance. ACS Symposium Series. 505: American Chemical Society; 1992. p. 31–40.

[45] Kasai S, Scott JG. Overexpression of cytochrome P450CYP6D1 is associated with monooxygenase-mediated pyrethroid resistance in house flies from Georgia. Pesticide Biochemistry and Physiology. 2000;68(1):34–41.

[46] Liu N, Scott JG. Increased transcription of CYP6D1 causes cytochrome P450 mediated insecticide resistance in house fly. Insect Biochemistry and Molecular Biology. 1998;28(8):531–5. 9753764 10.1016/s0965-1748(98)00039-3

[47] Kasai S, Scott JG. Expression and regulation of CYP6D3 in the house fly, Musca domestica (L.). Insect Biochemistry and Molecular Biology. 2001;32(1):1–8. 11719063 10.1016/s0965-1748(01)00073-x

[48] Daborn P, Boundy S, Yen J, Pittendrigh B, Ffrench-Constant R. DDT resistance in Drosophila correlates with Cyp6g1 over-expression and confers cross-resistance to the neonicotinoid imidacloprid. Molecular Genetics and Genomics. 2001;266(4):556–63. 10.1007/s004380100531 11810226

[49] Joussen N, Heckel DG, Haas M, Schuphan I, Schmidt B. Metabolism of imidacloprid and DDT by P450 CYP6G1 expressed in cell cultures of Nicotiana tabacum suggests detoxification of these insecticides in Cyp6g1-overexpressing strains of Drosophila melanogaster, leading to resistance. Pest Manag Sci. 2008;64(1):65–73. Epub 2007/10/04. 10.1002/ps.1472 17912692

[50] Gao Q, Li M, Sheng CF, Scott JG, Qiu XH. Multiple cytochrome P450s overexpressed in pyrethroid resistant house flies (Musca domestica). Pesticide Biochemistry and Physiology. 2012;104(3):252–60.

[51] Højland DH, Jensen K-MV, Kristensen M. Adaptation of Musca domestica L. Field Population to Laboratory Breeding Causes Transcriptional Alterations. PLoS ONE. 2014;9(1):e85965 10.1371/journal.pone.0085965 24489682 PMC3904851

[52] Zimmer CT, Bass C, Williamson MS, Kaussmann M, Wolfel K, Gutbrod O, et al Molecular and functional characterization of CYP6BQ23, a cytochrome P450 conferring resistance to pyrethroids in European populations of pollen beetle, Meligethes aeneus. Insect Biochem Mol Biol. 2014;45:18–29. 10.1016/j.ibmb.2013.11.008 24316412

[53] ffrench-Constant R, Daborn P, Feyereisen R. Resistance and the jumping gene. Bioessays. 2006;28(1):6–8. 10.1002/bies.20354 16369944

[54] Enayati AA, Ranson H, Hemingway J. Insect glutathione transferases and insecticide resistance. Insect Mol Biol. 2005;14(1):3–8. 10.1111/j.1365-2583.2004.00529.x 15663770

[55] Veal EA, Toone WM, Jones N, Morgan BA. Distinct roles for glutathione S-transferases in the oxidative stress response in Schizosaccharomyces pombe. J Biol Chem. 2002;277(38):35523–31. 10.1074/jbc.M111548200 12063243

[56] Kristensen M. Glutathione S-transferase and insecticide resistance in laboratory strains and field populations of Musca domestica. J Econ Entomol. 2005;98(4):1341–8. 16156589 10.1603/0022-0493-98.4.1341

[57] Shi J, Zhang L, Gao XW. Characterisation of spinosad resistance in the housefly Musca domestica (Diptera: Muscidae). Pest Management Science. 2011;67(3):335–40. 10.1002/ps.2073 21308959

[58] Kramer KJ, Hopkins TL. Tyrosine metabolism for insect cuticle tanning. Arch Insect Biochem. 1987;6(4):279–301.

[59] Bull DL, Whitten CJ. Factors influencing organophosphorus insecticide resistance in tobacco budworms. J Agric Food Chem. 1972;20(3):561–4. 5072307 10.1021/jf60181a061

[60] Epis S, Porretta D, Mastrantonio V, Urbanelli S, Sassera D, De Marco L, et al Temporal dynamics of the ABC transporter response to insecticide treatment: insights from the malaria vector Anopheles stephensi. Scientific Reports. 2014;4:7435 10.1038/srep07435 25504146 PMC4262823

[61] Orr N, Shaffner AJ, Richey K, Crouse GD. Novel mode of action of spinosad: Receptor binding studies demonstrating lack of interaction with known insecticidal target sites. Pestic Biochem Phys. 2009;95(1):1–5.

[62] Landis GN, Tower J. Superoxide dismutase evolution and life span regulation. Mechanisms of Ageing and Development. 2005;126(3):365–79. 10.1016/j.mad.2004.08.012 15664623

[63] Geng H, An C-J, Hao Y-J, Li D-S, Du R-Q. Molecular cloning and expression of Attacin from housefly (Musca domestica). Acta Genet Sin. 2004;31(12):1344–50. 15633638

[64] Mahmood K, Højland DH, Asp T, Kristensen M. Transcriptome Analysis of an Insecticide Resistant Housefly Strain: Insights about SNPs and Regulatory Elements in Cytochrome P450 Genes. PLoS ONE. 2016;11(3):e0151434 10.1371/journal.pone.0151434 27019205 PMC4809514

