# Supplementary material for: Analysis of Differentially Expressed Genes Related to Resistance in Spinosad- and Neonicotinoid-Resistant Musca domestica L. (Diptera: Muscidae) Strains
Source: PLoS One. 2017 Jan 26;12(1):e0170935. doi: 10.1371/journal.pone.0170935 (PMC5268453; doi:10.1371/journal.pone.0170935)
Supplement: S1 Table — Fold change (log2 values), logCPM, P-Value and FDR are provided. (PDF) [file pone.0170935.s001.pdf]

S1 Table. Raw data for global gene expression and expression of genes related to metabolism in the spinosad-resistant 791spin strain compared to the susceptible reference strain WHO-SRS. Fold change (log2 values), logCPM, P-Value and FDR are provided.

Gene	#feature	Description	logFC	logCPM	PValue	FDR
Global expression	LOC101899565	keratin-associated protein 19-2-like	10,131532	4,265236065	1,571E-09	2,04E-07
	LOC101889226	lysozyme 1-like	9,173488222	2,560026428	3,284E-12	1,1E-09
	LOC101894563	endocuticle structural glycoprotein SgAbd-2-like	8,896459314	2,467111878	1,602E-08	1,34E-06
	LOC101889416	uncharacterized	8,758898603	1,009540204	1,855E-10	3,27E-08
	LOC101888410	lysozyme 1-like	8,739782258	3,796230522	1,306E-13	5,03E-11
	LOC101900106	adult cuticle protein 1-like	8,110616687	5,358216965	3,509E-09	3,8E-07
	LOC101887361	adult cuticle protein 1-like	8,044513163	6,155523317	2,53E-10	4,38E-08
	LOC101897706	endocuticle structural glycoprotein SgAbd-5-like	8,016712486	7,549032576	3,426E-09	3,75E-07
	LOC101901361	uncharacterized	7,971904413	-0,720590084	2,253E-06	8,97E-05
	LOC101900178	adult cuticle protein 1-like	7,937367464	3,134714045	2,75E-07	1,51E-05
	LOC101899430	adult cuticle protein 1-like	7,865434618	4,121781535	3,767E-08	2,8E-06
	LOC101894425	cytochrome P450 4d8-like (4d63)	7,77416058	-0,287216575	1,76E-07	1,04E-05
	LOC101897260	probable serine threonine-protein kinase DDB_G0282963-like	7,742185222	-0,134140239	5,13E-09	5,42E-07
	LOC101901821	adult cuticle protein 1-like	7,720955962	7,511536196	2,953E-08	2,32E-06
	LOC101899637	acyl-CoA-binding protein-like	7,495141252	3,190183339	1,275E-19	2,21E-16
	LOC101900526	adult cuticle protein 1-like	7,451140907	7,579913754	5,492E-09	5,65E-07
	LOC101891902	pancreatic secretory trypsin inhibitor-like	7,436013362	3,201940758	8,036E-19	1,04E-15
	LOC101897557	uncharacterized	7,290620143	0,197011138	5,959E-12	1,88E-09
	LOC101894113	larval cuticle protein LCP-30-like	7,165309403	1,310184694	2,468E-12	8,55E-10
	LOC101900280	adult cuticle protein 1-like	7,135498083	5,247440478	5,573E-09	5,68E-07
	LOC101896247	uncharacterized	7,041195537	2,48790593	1,043E-10	2,17E-08
	LOC101900952	pupal cuticle protein-like	7,035140909	6,627531599	2,836E-08	2,25E-06
	LOC101893442	cuticle protein 1-like	6,867381934	7,481104984	9,233E-08	6,31E-06
P450	LOC101900728	cytochrome P450 18a1-like	-0,30081058	4,008818393	0,5175553	0,693185
	LOC101888518	cytochrome P450 302a1 mitochondrial-like	-0,63656241	4,821022347	0,0866191	0,251624
	LOC101901255	cytochrome P450 306a1-like	0,942724204	1,811248641	0,0894492	0,256076
	LOC101900639	cytochrome P450 307a1-like (307a2)	1,334715868	3,96219061	0,0071578	0,052048
	LOC101901025	cytochrome P450 315a1 mitochondrial-like	0,211845291	1,924133813	0,7451473	0,851609
	LOC101898177	cytochrome P450 4ae1-like (4ac3)	0,996947511	3,635333876	0,0848133	0,248613
	LOC101898930	cytochrome P450 4d1-like (4d3)	0,352105046	7,770311879	0,5859666	0,745626
	LOC101897841	cytochrome P450 4d2-like	1,346040622	5,113363035	0,0062294	0,046973
	LOC101898004	cytochrome P450 4d2-like	0,248180041	0,6617891	0,6982975	0,822304

LOC101897669	cytochrome P450 4d2-like (4d54)	-0,65557669	2,664095572	0,1947397	0,389442
LOC101892899	cytochrome P450 4d8-like	0,714050818	1,072812831	0,3628884	0,562041
LOC101894425	cytochrome P450 4d8-like (4d63)	7,77416058	-0,287216575	1,76E-07	1,04E-05
LOC101896081	cytochrome P450 4e2-like (4e11)	2,378393328	2,015986163	0,0004765	0,006973
LOC101895915	cytochrome P450 4e3-like (4e7)	0,743839854	5,25555101	0,2110642	0,408259
LOC101890640	cytochrome P450 4g15-like	0,705910279	2,161496836	0,2930565	0,495126
LOC101887550	cytochrome P450 4g1-like (4g13)	-5,37856591	7,519754654	1,39E-06	6,17E-05
LOC101887882	cytochrome P450 4g1-like (4g2)	0,64579705	10,9320273	0,3364156	0,537882
LOC101889105	cytochrome P450 4g1-like (4g98)	0,190768721	5,689572179	0,7893333	0,878248
LOC101891224	cytochrome P450 4p1-like (4p10)	1,43933013	4,872799345	0,0287919	0,136272
LOC101889365	cytochrome P450 6A1-like	2,497976783	1,11679531	0,0006664	0,009016
LOC101892246	cytochrome P450 6A1-like (6a24)	4,639302441	8,707048476	1,657E-13	6,15E-11
LOC101892072	cytochrome P450 6A1-like (6a57)	2,833725522	6,700588316	2,842E-08	2,25E-06
LOC101899899	cytochrome P450 6a2-like (6a54)	3,028193969	4,35540392	4,418E-06	0,000152
LOC101892970	cytochrome P450 6a8-like (6d8)	0,78861515	3,503869163	0,1730693	0,364189
LOC101894510	cytochrome P450 6a8-like (6gv1)	3,100583542	1,247629446	0,001245	0,014406
LOC101890041	cytochrome P450 6a9-like	1,814199634	4,115219601	0,0006175	0,008522
LOC101893114	cytochrome P450 6a9-like	1,404045863	2,295066134	0,0170985	0,096965
LOC101890373	cytochrome P450 6a9-like (6a37)	1,047513949	6,146887541	0,0117168	0,075386
LOC101891761	cytochrome P450 6a9-like (6a40)	1,20988754	3,352658946	0,0216235	0,11348
LOC101900791	cytochrome P450 6d1-like	3,039544301	8,605468613	4,026E-10	6,54E-08
LOC101889532	cytochrome P450 6d1-like	0,74650137	3,127304728	0,1232416	0,302886
LOC101899746	cytochrome P450 6d1-like	2,678149266	7,065558369	4,254E-06	0,000148
LOC101899135	cytochrome P450 6d1-like (6d12)	-1,09824771	1,557294224	0,084534	0,248204
LOC101899585	cytochrome P450 6d3-like	3,467048849	7,000535885	1,011E-08	9,2E-07
LOC101900431	cytochrome P450 6d3-like	1,758976238	1,654799051	0,0061471	0,046692
LOC101889269	cytochrome P450 6g1-like	-0,01465917	-0,01646483	1	1
LOC101898562	cytochrome P450 6g1-like (6g4)	2,306953061	8,614368556	1,585E-05	0,000443
LOC101889524	cytochrome P450 CYP12A2-like	2,710531199	5,131756052	2,364E-07	1,33E-05
LOC101889857	cytochrome P450 CYP12A2-like	1,920373989	4,468225444	0,0010146	0,012426
LOC101889684	cytochrome P450 CYP12A2-like	1,817912626	6,212497541	4,168E-05	0,000984
LOC101891274	cytochrome P450 CYP12A2-like	0,993462819	1,932905329	0,136006	0,32039
LOC101889672	cytochrome P450 CYP12A2-like	0,51238695	0,864038696	0,4545758	0,642784

LOC101890931	cytochrome P450 CYP12A2-like	0,404446718	3,163754574	0,4722201	0,657203
LOC101896195	cytochrome P450 CYP12A2-like	-0,03728923	3,840118868	0,9492685	0,974998
LOC101898453	cytochrome P450 CYP12A2-like (12a1)	5,215361942	3,520435115	1,604E-11	4,27E-09
LOC101890758	cytochrome P450 CYP12A2-like (12a1)	4,631563754	1,593763727	1,41E-07	8,83E-06
LOC101892495	probable cytochrome P450 12c1 mitochondrial-like	2,48159456	7,019335372	2,393E-07	1,34E-05
LOC101893522	probable cytochrome P450 12c1 mitochondrial-like (12g2)	4,609072963	7,527465737	2,136E-26	2,22E-22
LOC101900938	probable cytochrome P450 28a5-like	1,411590874	2,15419462	0,039151	0,16279
LOC101890714	probable cytochrome P450 28a5-like (28g6)	2,393620023	5,699682824	8,361E-06	0,00026
LOC101891587	probable cytochrome P450 28d1-like	1,493418497	4,162789347	0,0089976	0,062164
LOC101897279	probable cytochrome P450 28d1-like	2,4496637	6,718974331	4,255E-06	0,000148
LOC101897848	probable cytochrome P450 28d1-like	2,576583916	3,474614109	1,385E-06	6,17E-05
LOC101893000	probable cytochrome P450 301a1 mitochondrial-like	0,759671922	2,61673641	0,1783426	0,370205
LOC101895933	probable cytochrome P450 304a1-like	2,06298877	3,276885579	0,0028139	0,026366
LOC101889924	probable cytochrome P450 304a1-like	2,541118616	3,198484994	0,0003865	0,005932
LOC101890089	probable cytochrome P450 304a1-like	3,516095078	5,017430869	5,92E-09	5,91E-07
LOC101898997	probable cytochrome P450 305a1-like	1,847339089	2,995800422	0,0006017	0,008336
LOC101900906	probable cytochrome P450 308a1-like	1,628601819	7,429254511	0,0044594	0,03707
LOC101890335	probable cytochrome P450 309a2-like	0,740581945	3,883233144	0,3089841	0,510844
LOC101899919	probable cytochrome P450 310a1-like	0,721203387	0,499955933	0,3058271	0,507673
LOC101887394	probable cytochrome P450 311a1-like	0,915622703	3,274542389	0,1398906	0,323887
LOC101896297	probable cytochrome P450 313a4-like	0,500885178	0,760428586	0,6236242	0,772542
LOC101896469	probable cytochrome P450 313a4-like	0,957980658	6,05532499	0,1336591	0,317104
LOC101893162	probable cytochrome P450 313a4-like	1,518068602	3,568580409	0,0082665	0,058117
LOC101896650	probable cytochrome P450 313a4-like	1,667827169	2,866850177	0,0071028	0,05172
LOC101890728	probable cytochrome P450 313a4-like (313d1)	5,441523621	0,872297527	5,431E-06	0,000181
LOC101891061	probable cytochrome P450 317a1-like	1,317597396	3,576412259	0,02219	0,115751
LOC101892636	probable cytochrome P450 318a1-like	1,169921124	2,677835388	0,0619009	0,209107
LOC101887655	probable cytochrome P450 49a1-like	0,756468894	1,337073953	0,2411614	0,440948
LOC101901643	probable cytochrome P450 4ac1-like	1,594959824	4,184763013	0,0275465	0,132701
LOC101897760	probable cytochrome P450 4ad1-like	1,977113634	3,678301776	0,0003084	0,004983
LOC101897033	probable cytochrome P450 4d14-like	1,826389606	0,584512002	0,0048628	0,039261
LOC101891759	probable cytochrome P450 4d14-like	2,087518884	6,253726954	4,403E-05	0,001026
LOC101891931	probable cytochrome P450 4d14-like	1,720179262	3,824893101	0,0014451	0,016181

LOC101897209	probable cytochrome P450 4d14-like (4d9)	5,149691663	3,963049199	1,192E-11	3,35E-09
LOC101893472	probable cytochrome P450 4p3-like	2,512930362	4,1562985	1,8E-05	0,000492
LOC101891157	probable cytochrome P450 4s3-like	2,722903732	4,614896092	8,162E-09	7,78E-07
LOC101892717	probable cytochrome P450 4s3-like (4s23)	-0,98824435	3,372344084	0,0426453	0,169911
LOC101895233	probable cytochrome P450 4aa1-like	-0,11545529	2,297214001	0,8915533	0,941916
LOC101891933	probable cytochrome P450 6a13-like	2,194292366	3,885209247	4,183E-05	0,000986
LOC101890889	probable cytochrome P450 6a14-like	-0,06678539	3,683963638	0,905832	0,949834
LOC101890715	probable cytochrome P450 6a14-like	1,117876245	4,260996954	0,0468339	0,178784
LOC101892622	probable cytochrome P450 6a14-like	1,175174967	0,971846293	0,095736	0,26526
LOC101892108	probable cytochrome P450 6a14-like	1,301739271	0,213480441	0,0795835	0,239762
LOC101892278	probable cytochrome P450 6a14-like	1,953407807	1,412089871	0,001684	0,018059
LOC101892447	probable cytochrome P450 6a14-like	2,01743813	1,426789691	0,0007054	0,009374
LOC101900065	probable cytochrome P450 6a14-like	2,907615339	6,939905898	1,496E-05	0,000422
LOC101887482	probable cytochrome P450 6a17-like	1,672782606	1,022512018	0,0062281	0,046973
LOC101890543	probable cytochrome P450 6a17-like (6gu1)	3,27443669	2,157875742	1,225E-05	0,00036
LOC101898668	probable cytochrome P450 6a18-like	1,417978089	4,559979567	0,0012546	0,014502
LOC101892931	probable cytochrome P450 6a21-like	-0,08453743	4,224926286	0,8844943	0,938003
LOC101889704	probable cytochrome P450 6a21-like	0,200987601	1,266249008	0,7626618	0,863552
LOC101891408	probable cytochrome P450 6a21-like	0,348373869	1,829744447	0,58667	0,745973
LOC101895803	probable cytochrome P450 6a21-like	2,586149046	4,304983772	1,723E-05	0,000474
LOC101892586	probable cytochrome P450 6a21-like	2,984568695	3,578678339	5,744E-07	2,88E-05
LOC101889539	probable cytochrome P450 6a21-like (6a36)	1,919455654	3,009384278	0,0006684	0,009031
LOC101890199	probable cytochrome P450 6a21-like (6a37)	1,197639511	4,376832477	0,0118697	0,076017
LOC101891297	probable cytochrome P450 6d5-like	1,135307763	6,978798192	0,1615978	0,351189
LOC101900444	probable cytochrome P450 6g2-like	-0,78002152	-0,189906625	0,5472729	0,715093
LOC101899434	probable cytochrome P450 6t3-like	1,613260591	-0,203460148	0,0584223	0,202761
LOC101887226	probable cytochrome P450 6u1-like	0,359650081	4,962336251	0,3340059	0,536009
LOC101896204	probable cytochrome P450 6v1-like	0,018784979	4,811743365	0,9602778	0,98047
LOC101898942	probable cytochrome P450 9f2-like	-0,72898879	3,421413896	0,1914127	0,385534
LOC101898775	probable cytochrome P450 9f2-like	2,349403889	3,215512471	2,302E-05	0,000606
LOC101898478	probable cytochrome P450 9f2-like	1,119207965	7,641641298	0,0282333	0,134998
LOC101899118	probable cytochrome P450 9f2-like (9f11)	-2,27101029	2,680234948	0,00054	0,007656
LOC101900658	probable cytochrome P450 9f2-like (9f12)	3,596184208	4,311916412	1,669E-08	1,37E-06

GST	LOC101897621	glutathione S-transferase 1-1-like	5,963281698	5,544765685	7,072E-25	3,67E-21
	LOC101895956	glutathione S-transferase 1-1-like	1,14306142	4,552460566	0,010038	0,06738
	LOC101895036	glutathione S-transferase 1-like	4,383367086	6,465701061	1,694E-17	1,35E-14
	LOC101900016	glutathione S-transferase 1-like	2,767674451	3,883061346	1,843E-07	1,08E-05
	LOC101888349	glutathione S-transferase 1-like	2,418470921	1,409576208	0,0001455	0,002738
	LOC101887423	glutathione S-transferase 1-like	2,282367279	8,538829639	6,879E-06	0,000222
	LOC101887250	glutathione S-transferase 1-like	1,766673463	7,100119283	0,0009658	0,011947
	LOC101895555	glutathione S-transferase 1-like	1,204330182	8,152821828	0,018784	0,103326
	LOC101899848	glutathione S-transferase 1-like	0,95261242	3,954983238	0,0624415	0,210176
	LOC101900672	glutathione S-transferase 1-like	0,740241202	7,795008073	0,0728385	0,228082
	LOC101888181	glutathione S-transferase 1-like	0,070418997	1,038355947	0,9386554	0,969867
	LOC101894873	glutathione S-transferase 1-like	-0,794493	7,454510001	0,1126463	0,288231
	LOC101895607	glutathione S-transferase 1-like	-1,56104848	5,232596968	2,011E-05	0,00054
	LOC101895316	glutathione S-transferase 1-like	0,134357653	7,575250148	0,7384484	0,846629
	LOC101897277	glutathione S-transferase 2-like	2,228189726	5,479178237	4,078E-07	2,13E-05
	LOC101897094	glutathione S-transferase 2-like	1,217680066	4,679604074	0,0040919	0,034794
	LOC101897797	glutathione S-transferase D7-like	1,775654652	0,357353549	0,0220926	0,115359
	LOC101891696	glutathione S-transferase omega-1-like	0,146701946	6,260660658	0,6471054	0,787342
	LOC101900949	glutathione S-transferase theta-1-like	1,030261713	5,11414214	0,0038661	0,033338
	LOC101897781	glutathione S-transferase theta-1-like	0,938641967	5,282329244	0,0390981	0,16279
	LOC101888110	glutathione S-transferase theta-1-like	0,70961036	2,295259512	0,2343248	0,434798
	LOC101890402	glutathione S-transferase theta-1-like	0,316156522	2,350994381	0,6134589	0,764661
	LOC101898455	glutathione S-transferase theta-1-like	-0,00681288	2,58639472	0,9949893	0,999743
	LOC101890455	glutathione S-transferase-like	1,873426123	6,616672985	0,0002154	0,003749
Esterase	LOC101900490	esterase B1-like	2,000758059	3,934793336	0,000403	0,006132
	LOC101896978	esterase B1-like	1,952180991	7,212302707	3,053E-05	0,000759
	LOC101896625	esterase B1-like	1,666596461	8,417309364	0,0041743	0,035219
	LOC101896807	esterase B1-like	1,633594153	6,597998298	9,768E-05	0,001975
	LOC101898526	esterase B1-like	1,524930993	3,858570583	0,0291939	0,137016
	LOC101896445	esterase B1-like	1,218229405	3,906292112	0,0147794	0,088108
	LOC101895121	esterase B1-like	1,166428522	3,211856925	0,062683	0,210721
	LOC101898347	esterase B1-like	1,144170679	4,494798263	0,0447542	0,174368
	LOC101898354	esterase B1-like	1,039622226	1,437774926	0,1138659	0,289357

	LOC101897154	esterase B1-like	0,665963129	5,743012087	0,1566213	0,345458
	LOC101897334	esterase B1-like	0,587716022	4,683493429	0,3410515	0,542456
	LOC101897501	esterase B1-like	0,419701887	5,611585153	0,2251208	0,424081
	LOC101889275	esterase B1-like	-0,2505826	4,714909099	0,507006	0,684995
	LOC101898698	esterase B1-like	-0,38056742	2,9763668	0,6558251	0,792475
	LOC101890018	esterase FE4-like	1,865537078	2,889402769	0,0050172	0,040164
	LOC101889364	esterase-5A-like	1,087056685	6,332141317	0,0265749	0,129826
UGT	LOC101888811	UDP-glucuronosyltransferase 2A2-like	1,72429586	3,385124693	0,0038098	0,032962
	LOC101893116	UDP-glucuronosyltransferase 2A3-like	3,339326506	4,899949527	1,754E-09	2,2E-07
	LOC101893291	UDP-glucuronosyltransferase 2A3-like	2,174765445	6,540181742	4,585E-06	0,000156
	LOC101889193	UDP-glucuronosyltransferase 2A3-like	2,231442169	4,256032423	6,678E-05	0,001446
	LOC101889322	UDP-glucuronosyltransferase 2B13-like	3,067678909	6,01009183	1,833E-10	3,27E-08
	LOC101890271	UDP-glucuronosyltransferase 2B13-like	2,161978562	4,612746069	1,063E-05	0,000318
	LOC101900184	UDP-glucuronosyltransferase 2B13-like	1,184927494	4,881741127	0,0032571	0,029151
	LOC101895816	UDP-glucuronosyltransferase 2B15-like	2,027754206	5,716331269	0,000109	0,002166
	LOC101897252	UDP-glucuronosyltransferase 2B15-like	1,438258569	1,532907397	0,0120606	0,076656
	LOC101899999	UDP-glucuronosyltransferase 2B17-like	0,795914459	7,542089237	0,035436	0,153423
	LOC101899504	UDP-glucuronosyltransferase 2B17-like	0,465128453	4,745432103	0,2657654	0,466876
	LOC101893458	UDP-glucuronosyltransferase 2B1-like	2,203750589	4,818435119	0,0033309	0,029633
	LOC101897074	UDP-glucuronosyltransferase 2B1-like	0,923083296	3,532212992	0,1419654	0,326725
	LOC101890444	UDP-glucuronosyltransferase 2B20-like	1,041456186	4,493935334	0,0394177	0,163257
	LOC101890612	UDP-glucuronosyltransferase 2B20-like	0,652263394	3,86960014	0,2247341	0,42366
	LOC101892660	UDP-glucuronosyltransferase 2B31-like	1,016794502	5,727941067	0,0192213	0,104789
	LOC101890269	UDP-glucuronosyltransferase 2B33-like	1,447385122	3,412502334	0,0064675	0,048313
	LOC101890707	UDP-glucuronosyltransferase 2B4-like	-0,46515303	0,161243907	0,5358134	0,707013
	LOC101889773	UDP-glucuronosyltransferase 2B7-like	2,015714502	4,382231096	0,0001256	0,002396
	LOC101893619	UDP-glucuronosyltransferase 2B9-like	-0,66111354	0,967780928	0,4006715	0,594938
	LOC101889496	UDP-glucuronosyltransferase 2C1-like	1,530754903	3,61982905	0,0048713	0,0393
	LOC101889147	UDP-glucuronosyltransferase-like	5,60500912	2,905539956	8,713E-14	3,48E-11
	LOC101893798	UDP-glucuronosyltransferase-like	-0,40959197	5,254465421	0,2410393	0,440849
	LOC101892938	UDP-glucuronosyltransferase-like	-1,40612819	3,405771085	0,0140771	0,085119
	LOC101899202	UDP-glucuronosyltransferase-like	-1,61902492	2,372905438	0,0070683	0,051577
	LOC101899032	UDP-glucuronosyltransferase-like	-2,50564081	2,971496127	2,344E-05	0,000614

	LOC101892765	UDP-glucuronosyltransferase-like	-2,51798458	-0,03180039	0,0017463	0,018527
ABC	LOC101895261	ABC transporter F family member 4-like	-0,43839904	5,354639716	0,2723703	0,47415
	LOC101892798	ABC transporter G family member 1-like	-0,11817213	2,911271799	0,8295074	0,903218
	LOC101895448	ABC transporter G family member 20-like	1,44652234	4,041618078	0,0322375	0,145202
	LOC101889472	ABC transporter G family member 20-like	1,737143313	5,394445632	0,0016348	0,017622
	LOC101898054	ABC transporter G family member 20-like	0,877585331	5,485663165	0,0524643	0,189752
	LOC101890903	ABC transporter G family member 22-like	-0,8440581	6,031402911	0,0371164	0,157419
	LOC101899230	ATP-binding cassette sub-family A member 13-like	-0,28653219	5,729211924	0,3860635	0,581558
	LOC101888352	ATP-binding cassette sub-family A member 3-like	0,581404672	6,842363526	0,2212768	0,420038
	LOC101895192	ATP-binding cassette sub-family B member 10 mitochondrial-like	-0,52523441	5,335480901	0,137329	0,321615
	LOC101899501	ATP-binding cassette sub-family B member 6 mitochondrial-like	-0,49026368	6,22456283	0,136352	0,320914
	LOC101888322	ATP-binding cassette sub-family B member 7 mitochondrial-like	-0,08821753	5,7493653	0,7766252	0,870653
	LOC101888861	ATP-binding cassette sub-family B member 7 mitochondrial-like	-0,70184093	2,54679236	0,1737562	0,365116
	LOC101891270	ATP-binding cassette sub-family B member 8 mitochondrial-like	0,066682297	5,949297353	0,8275733	0,902105
	LOC101896101	ATP-binding cassette sub-family C member Sur-like	-0,52041956	2,524809115	0,382023	0,578238
	LOC101887509	ATP-binding cassette sub-family D member 2-like	0,123667637	5,75693345	0,7308228	0,842508
	LOC101901316	ATP-binding cassette sub-family D member 3-like	0,327858797	6,932821961	0,3871071	0,582381
	LOC101894209	ATP-binding cassette sub-family E member 1-like	-0,55188128	8,793166779	0,1327024	0,315757
	LOC101901102	ATP-binding cassette sub-family F member 1-like	0,186988803	7,435114771	0,6086488	0,762282
	LOC101897819	ATP-binding cassette sub-family F member 2-like	-0,31301439	8,837344729	0,403305	0,597312
	LOC101901338	ATP-binding cassette sub-family F member 3-like	-0,37390831	6,580129729	0,2720579	0,473831
	LOC101894746	ATP-binding cassette sub-family G member 1-like	1,965894819	2,834556957	0,0036219	0,031653
	LOC101897386	ATP-binding cassette sub-family G member 1-like	1,546222612	3,108324966	0,0149098	0,08853
	LOC101894584	ATP-binding cassette sub-family G member 1-like	1,235846073	5,540785046	0,0233824	0,119846
	LOC101897551	ATP-binding cassette sub-family G member 1-like	0,970143326	7,135374516	0,0648065	0,214404
	LOC101897724	ATP-binding cassette sub-family G member 1-like	0,471725967	4,187040751	0,432829	0,62509
	LOC101888695	ATP-binding cassette sub-family G member 1-like	-0,14034231	5,916190315	0,7083328	0,828115
	LOC101896421	ATP-binding cassette sub-family G member 1-like	-0,95628988	5,16961226	0,0144728	0,086679
	LOC101890630	ATP-binding cassette sub-family G member 4-like	2,179823974	2,213822518	0,0012087	0,014081
	LOC101897891	ATP-binding cassette sub-family G member 4-like	1,322564549	0,897226221	0,0886116	0,255059
	LOC101890462	ATP-binding cassette sub-family G member 4-like	1,165350002	4,30821233	0,0303256	0,140581
	LOC101894909	ATP-binding cassette sub-family G member 4-like	0,908649797	4,055415828	0,1300696	0,312353
	LOC101887291	ATP-binding cassette sub-family G member 4-like	0,794957909	5,797313455	0,0239788	0,12164

LOC101899158	ATP-binding cassette sub-family G member 5-like	1,019136436	2,855070399	0,0691726	0,221981
