# Supplementary material for: Analysis of Differentially Expressed Genes Related to Resistance in Spinosad- and Neonicotinoid-Resistant Musca domestica L. (Diptera: Muscidae) Strains
Source: PLoS One. 2017 Jan 26;12(1):e0170935. doi: 10.1371/journal.pone.0170935 (PMC5268453; doi:10.1371/journal.pone.0170935)
Supplement: S2 Table — Fold change (log2 values), logCPM, P-Value and FDR are provided. (PDF) [file pone.0170935.s002.pdf]

S2 Table. Raw data for global gene expression and expression of genes related to metabolism in the neonicotinoid-resistant 766b strain compared to the susceptible reference strain WHO-SRS. Fold change (log2 values), logCPM, P-Value and FDR are provided.

Gene	#feature	description	logFC	logCPM	PValue	FDR
Global expression	LOC101889416	uncharacterized	9,24148	1,009540204	0,000	2,59787E-09
	LOC101899031	desumoylating isopeptidase 2-like	8,53036	-0,352521041	0,000	1,78502E-08
	LOC101897260	probable serine threonine-protein kinase DDB_G0282963-like	7,91744	-0,134140239	0,000	2,29949E-07
	LOC101891094	pupal cuticle protein C1B-like	7,83707	0,687094665	0,000	8,54314E-10
	LOC101899799	long-chain-fatty-acid--CoA ligase bubblegum-like	7,71837	0,560058176	0,000	4,31528E-11
	LOC101891902	pancreatic secretory trypsin inhibitor-like	7,67427	3,201940758	0,000	2,51988E-16
	LOC101894425	cytochrome P450 4d8-like (4d63)	7,57023	-0,287216575	0,000	3,28904E-05
	LOC101897815	uncharacterized	7,46686	2,232874797	0,000	8,20753E-14
	LOC101893653	plasminogen-like	7,32201	4,813188891	0,000	3,50782E-16
	LOC101900763	membrane-bound alkaline phosphatase-like	7,06287	1,456937512	0,000	4,34341E-12
	LOC101894897	uncharacterized	7,06086	0,69592017	0,000	7,27984E-06
	LOC101894113	larval cuticle protein LCP-30-like	6,32283	1,310184694	0,000	1,37472E-07
	LOC101900952	pupal cuticle protein-like	6,25860	6,627531599	0,000	3,00522E-05
	LOC101894563	endocuticle structural glycoprotein SgAbd-2-like	5,97754	2,467111878	0,000	0,002332105
	LOC101888737	uncharacterized	5,81383	0,089725382	0,000	3,1112E-07
	LOC101892246	cytochrome P450 6A1-like (6a24)	5,56325	8,707048476	0,000	2,20419E-14
	LOC101900280	adult cuticle protein 1-like	5,55436	5,247440478	0,000	8,92695E-05
	LOC101900526	adult cuticle protein 1-like	5,51895	7,579913754	0,000	0,000178771
	LOC101892465	uncharacterized	5,50533	1,884652195	0,000	8,88751E-10
	LOC101901361	uncharacterized	5,48146	-0,720590084	0,004	0,046477027
	LOC101889243	membrane metallo-endopeptidase-like 1-like	5,48114	1,432928363	0,000	3,44778E-09
P450	LOC101900728	cytochrome P450 18a1-like	0,60010	4,008818393	0,193	0,397191007
	LOC101888518	cytochrome P450 302a1 mitochondrial-like	-0,60498	4,821022347	0,103	0,291297953
	LOC101901255	cytochrome P450 306a1-like	0,72811	1,811248641	0,189	0,392186886
	LOC101900639	cytochrome P450 307a1-like	0,78192	3,96219061	0,111	0,302116128
	LOC101901025	cytochrome P450 315a1 mitochondrial-like	1,38252	1,924133813	0,035	0,171362913
	LOC101898177	cytochrome P450 4ae1-like	1,21694	3,635333876	0,036	0,174550372
	LOC101898930	cytochrome P450 4d1-like	1,78981	7,770311879	0,007	0,066275346
	LOC101898004	cytochrome P450 4d2-like	-0,36761	0,6617891	0,545	0,708633003
	LOC101897669	cytochrome P450 4d2-like (4d54)	-1,09674	2,664095572	0,030	0,157064312
	LOC101897841	cytochrome P450 4d2-like (4d56)	-1,33625	5,113363035	0,007	0,06523937
	LOC101892899	cytochrome P450 4d8-like	0,42127	1,072812831	0,593	0,746773447

LOC101894425	cytochrome P450 4d8-like (4d63)	7,57023	-0,287216575	0,000	3,28904E-05
LOC101896081	cytochrome P450 4e2-like	1,29742	2,015986163	0,052	0,210159441
LOC101895915	cytochrome P450 4e3-like	1,87225	5,25555101	0,002	0,029508552
LOC101890640	cytochrome P450 4g15-like	0,92151	2,161496836	0,168	0,368323029
LOC101887550	cytochrome P450 4g1-like (4g13)	-2,70141	7,519754654	0,006	0,060991306
LOC101887882	cytochrome P450 4g1-like (4g2)	1,82383	10,9320273	0,008	0,072150303
LOC101889105	cytochrome P450 4g1-like (4g98)	3,20348	5,689572179	0,000	0,001287729
LOC101891224	cytochrome P450 4p1-like (4p10)	2,45049	4,872799345	0,000	0,007548395
LOC101889365	cytochrome P450 6A1-like	1,65541	1,11679531	0,023	0,136147747
LOC101892246	cytochrome P450 6A1-like (6a24)	5,56325	8,707048476	0,000	2,20419E-14
LOC101892072	cytochrome P450 6A1-like (6a57)	4,21344	6,700588316	0,000	4,34341E-12
LOC101899899	cytochrome P450 6a2-like	1,28677	4,35540392	0,039	0,181738188
LOC101892970	cytochrome P450 6a8-like	1,31372	3,503869163	0,024	0,139971367
LOC101894510	cytochrome P450 6a8-like (6gv1)	3,82688	1,247629446	0,000	0,002831736
LOC101893114	cytochrome P450 6a9-like	0,72523	2,295066134	0,211	0,417741958
LOC101890041	cytochrome P450 6a9-like	0,54756	4,115219601	0,291	0,497092557
LOC101891761	cytochrome P450 6a9-like	0,18436	3,352658946	0,729	0,842185885
LOC101890373	cytochrome P450 6a9-like (6a37)	-0,02525	6,146887541	0,952	0,978025746
LOC101899746	cytochrome P450 6d1-like	2,07535	7,065558369	0,000	0,006782543
LOC101900791	cytochrome P450 6d1-like	1,93195	8,605468613	0,000	0,001447486
LOC101889532	cytochrome P450 6d1-like	0,30215	3,127304728	0,532	0,698543403
LOC101899135	cytochrome P450 6d1-like (6d12)	-1,51140	1,557294224	0,017	0,115003371
LOC101899585	cytochrome P450 6d3-like	4,53422	7,000535885	0,000	3,53238E-10
LOC101900431	cytochrome P450 6d3-like	1,08433	1,654799051	0,089	0,274157529
LOC101889269	cytochrome P450 6g1-like	-0,09794	-0,01646483	0,926	0,962453958
LOC101898562	cytochrome P450 6g1-like (6g4)	3,35455	8,614368556	0,000	3,45721E-07
LOC101889857	cytochrome P450 CYP12A2-like	2,43119	4,468225444	0,000	0,001690999
LOC101889684	cytochrome P450 CYP12A2-like	1,24828	6,212497541	0,004	0,047027098
LOC101891274	cytochrome P450 CYP12A2-like	1,14156	1,932905329	0,083	0,266016842
LOC101896195	cytochrome P450 CYP12A2-like	0,87405	3,840118868	0,090	0,27545872
LOC101890931	cytochrome P450 CYP12A2-like	0,13386	3,163754574	0,814	0,89770655
LOC101889524	cytochrome P450 CYP12A2-like	-0,05861	5,131756052	0,908	0,953590773
LOC101889672	cytochrome P450 CYP12A2-like	-0,40914	0,864038696	0,573	0,730480315

LOC101898453	cytochrome P450 CYP12A2-like (12a1)	2,78162	3,520435115	0,000	0,002847893
LOC101890758	cytochrome P450 CYP12A2-like (12a1)	2,73568	1,593763727	0,001	0,016541731
LOC101892495	probable cytochrome P450 12c1 mitochondrial-like	1,52189	7,019335372	0,001	0,018406785
LOC101893522	probable cytochrome P450 12c1 mitochondrial-like (12g2)	5,05660	7,527465737	0,000	1,04584E-26
LOC101900938	probable cytochrome P450 28a5-like	1,40188	2,15419462	0,040	0,18484177
LOC101890714	probable cytochrome P450 28a5-like	1,30436	5,699682824	0,012	0,093519258
LOC101897848	probable cytochrome P450 28d1-like	1,20768	3,474614109	0,019	0,121705019
LOC101891587	probable cytochrome P450 28d1-like	1,19318	4,162789347	0,035	0,173187202
LOC101897279	probable cytochrome P450 28d1-like (28b1)	2,60658	6,718974331	0,000	8,65228E-05
LOC101893000	probable cytochrome P450 301a1 mitochondrial-like	1,17781	2,61673641	0,038	0,178758558
LOC101890089	probable cytochrome P450 304a1-like	1,31254	5,017430869	0,019	0,122386947
LOC101889924	probable cytochrome P450 304a1-like	1,01253	3,198484994	0,143	0,339569941
LOC101895933	probable cytochrome P450 304a1-like	0,52009	3,276885579	0,439	0,62536046
LOC101898997	probable cytochrome P450 305a1-like	0,97586	2,995800422	0,066	0,236638544
LOC101900906	probable cytochrome P450 308a1-like	1,93234	7,429254511	0,001	0,015164551
LOC101890335	probable cytochrome P450 309a2-like (437a4)	2,23617	3,883233144	0,003	0,036777156
LOC101899919	probable cytochrome P450 310a1-like	1,90694	0,499955933	0,005	0,052516624
LOC101887394	probable cytochrome P450 311a1-like	2,01638	3,274542389	0,001	0,02208428
LOC101893162	probable cytochrome P450 313a4-like	1,87274	3,568580409	0,001	0,019666712
LOC101896650	probable cytochrome P450 313a4-like	1,80364	2,866850177	0,004	0,04177385
LOC101896469	probable cytochrome P450 313a4-like	-0,26092	6,05532499	0,681	0,808410526
LOC101896297	probable cytochrome P450 313a4-like	-0,57963	0,760428586	0,579	0,735400368
LOC101890728	probable cytochrome P450 313a4-like (313d1)	3,73792	0,872297527	0,001	0,022286857
LOC101891061	probable cytochrome P450 317a1-like	0,89727	3,576412259	0,116	0,309095807
LOC101892636	probable cytochrome P450 318a1-like	0,89603	2,677835388	0,150	0,348638644
LOC101887655	probable cytochrome P450 49a1-like	0,26843	1,337073953	0,680	0,80819226
LOC101901643	probable cytochrome P450 4ac1-like (4ac6)	2,81792	4,184763013	0,000	0,005211481
LOC101897760	probable cytochrome P450 4ad1-like	0,30898	3,678301776	0,567	0,726394756
LOC101897033	probable cytochrome P450 4d14-like	1,75292	0,584512002	0,007	0,065845063
LOC101891931	probable cytochrome P450 4d14-like	1,31632	3,824893101	0,014	0,100085348
LOC101891759	probable cytochrome P450 4d14-like (4d4)	3,01379	6,253726954	0,000	1,89891E-06
LOC101897209	probable cytochrome P450 4d14-like (4d9)	2,09191	3,963049199	0,002	0,028864371
LOC101893472	probable cytochrome P450 4p3-like	1,45623	4,1562985	0,010	0,084774546

	LOC101891157	probable cytochrome P450 4s3-like	1,85647	4,614896092	0,000	0,002014269
	LOC101892717	probable cytochrome P450 4s3-like	-0,61427	3,372344084	0,204	0,409075138
	LOC101895233	probable cytochrome P450 4aa1-like	0,81513	2,297214001	0,278	0,484752634
	LOC101891933	probable cytochrome P450 6a13-like	0,76781	3,885209247	0,138	0,33379806
	LOC101892622	probable cytochrome P450 6a14-like	1,40702	0,971846293	0,045	0,1948088
	LOC101890715	probable cytochrome P450 6a14-like	1,11369	4,260996954	0,047	0,200314083
	LOC101892278	probable cytochrome P450 6a14-like	0,72989	1,412089871	0,235	0,441934911
	LOC101892447	probable cytochrome P450 6a14-like	0,52405	1,426789691	0,367	0,564112807
	LOC101890889	probable cytochrome P450 6a14-like	0,24724	3,683963638	0,643	0,784450974
	LOC101900065	probable cytochrome P450 6a14-like	0,13222	6,939905898	0,834	0,908978259
	LOC101892108	probable cytochrome P450 6a14-like	0,10327	0,213480441	0,915	0,957258189
	LOC101890543	probable cytochrome P450 6a17-like	1,72528	2,157875742	0,016	0,111089567
	LOC101887482	probable cytochrome P450 6a17-like	1,14688	1,022512018	0,057	0,220786124
	LOC101898668	probable cytochrome P450 6a18-like	0,25730	4,559979567	0,553	0,715261145
	LOC101892586	probable cytochrome P450 6a21-like	1,96393	3,578678339	0,001	0,012827254
	LOC101892931	probable cytochrome P450 6a21-like (6a25)	-1,48733	4,224926286	0,010	0,084801859
	LOC101889539	probable cytochrome P450 6a21-like (6a36)	0,38917	3,009384278	0,481	0,660384208
	LOC101890199	probable cytochrome P450 6a21-like (6a37)	0,33178	4,376832477	0,483	0,661116256
	LOC101895803	probable cytochrome P450 6a21-like (6a56)	2,12265	4,304983772	0,000	0,007847971
	LOC101891408	probable cytochrome P450 6a21-like (6a58)	-1,65579	1,829744447	0,012	0,090908786
	LOC101889704	probable cytochrome P450 6a21-like (6a7)	-2,70106	1,266249008	0,000	0,004515596
	LOC101891297	probable cytochrome P450 6d5-like (6d8)	2,95433	6,978798192	0,001	0,012326031
	LOC101900444	probable cytochrome P450 6g2-like (6g7)	2,51375	-0,189906625	0,007	0,064582029
	LOC101899434	probable cytochrome P450 6t3-like	1,98174	-0,203460148	0,018	0,117557891
	LOC101887226	probable cytochrome P450 6u1-like	0,49013	4,962336251	0,188	0,391160526
	LOC101896204	probable cytochrome P450 6v1-like	0,36676	4,811743365	0,313	0,518540399
	LOC101900658	probable cytochrome P450 9f2-like	1,92923	4,311916412	0,001	0,020687381
	LOC101898942	probable cytochrome P450 9f2-like	0,45927	3,421413896	0,403	0,595275722
	LOC101899118	probable cytochrome P450 9f2-like	-0,65496	2,680234948	0,289	0,494817273
	LOC101898478	probable cytochrome P450 9f2-like	1,45153	7,641641298	0,005	0,050857323
	LOC101898775	probable cytochrome P450 9f2-like (9f7)	2,88285	3,215512471	0,000	2,89893E-05
GST	LOC101897212	glutathione S-transferase 1 isoform C-like	0,33311	3,361457006	0,490	0,66658532
	LOC101897621	glutathione S-transferase 1-1-like	1,68764	5,544765685	0,001	0,012859491

	LOC101895956	glutathione S-transferase 1-1-like	0,48157	4,552460566	0,275	0,482105846
	LOC101895036	glutathione S-transferase 1-like	3,13996	6,465701061	0,000	2,65588E-08
	LOC101900016	glutathione S-transferase 1-like	2,42997	3,883061346	0,000	0,000220935
	LOC101888349	glutathione S-transferase 1-like	1,46924	1,409576208	0,021	0,127468941
	LOC101887423	glutathione S-transferase 1-like	0,20387	8,538829639	0,674	0,804556686
	LOC101899848	glutathione S-transferase 1-like	0,11212	3,954983238	0,825	0,904464785
	LOC101900672	glutathione S-transferase 1-like	-0,06410	7,795008073	0,876	0,934837624
	LOC101887250	glutathione S-transferase 1-like	-0,08929	7,100119283	0,864	0,927840332
	LOC101894873	glutathione S-transferase 1-like	-0,43274	7,454510001	0,385	0,57951614
	LOC101895555	glutathione S-transferase 1-like	-0,84892	8,152821828	0,095	0,281946114
	LOC101888181	glutathione S-transferase 1-like	-1,40392	1,038355947	0,066	0,237236961
	LOC101895607	glutathione S-transferase 1-like	-2,07431	5,232596968	0,000	3,20997E-06
	LOC101895316	glutathione S-transferase 1-like	0,52908	7,575250148	0,189	0,39233527
	LOC101897094	glutathione S-transferase 2-like	1,70232	4,679604074	0,000	0,002598634
	LOC101897277	glutathione S-transferase 2-like	0,88953	5,479178237	0,037	0,175883612
	LOC101897797	glutathione S-transferase D7-like	0,29162	0,357353549	0,719	0,835038331
	LOC101891696	glutathione S-transferase omega-1-like	-0,00575	6,260660658	0,986	0,994820327
	LOC101897781	glutathione S-transferase theta-1-like	0,88433	5,282329244	0,052	0,21028165
	LOC101900949	glutathione S-transferase theta-1-like	0,41757	5,11414214	0,239	0,445998168
	LOC101890402	glutathione S-transferase theta-1-like	-0,42221	2,350994381	0,492	0,668242061
	LOC101898455	glutathione S-transferase theta-1-like	-0,97780	2,58639472	0,075	0,253327337
	LOC101888110	glutathione S-transferase theta-1-like	-1,33832	2,295259512	0,028	0,151368553
	LOC101890455	glutathione S-transferase-like	1,86612	6,616672985	0,000	0,006118319
Esterase	LOC101898526	esterase B1-like	2,49780	3,858570583	0,001	0,01121752
	LOC101896625	esterase B1-like	2,07409	8,417309364	0,000	0,009651192
	LOC101898354	esterase B1-like	2,05256	1,437774926	0,002	0,026435601
	LOC101898698	esterase B1-like	1,97769	2,9763668	0,021	0,129337004
	LOC101895121	esterase B1-like	1,96287	3,211856925	0,002	0,028779854
	LOC101898347	esterase B1-like	1,78036	4,494798263	0,002	0,029021301
	LOC101900490	esterase B1-like	1,68113	3,934793336	0,003	0,034397067
	LOC101896978	esterase B1-like	1,46181	7,212302707	0,002	0,023747398
	LOC101897154	esterase B1-like	1,43252	5,743012087	0,003	0,033946392
	LOC101896807	esterase B1-like	1,20154	6,597998298	0,004	0,043135588

	LOC101897334	esterase B1-like	1,16884	4,683493429	0,060	0,226967108
	LOC101896445	esterase B1-like	0,29942	3,906292112	0,544	0,708425999
	LOC101897501	esterase B1-like	0,23469	5,611585153	0,498	0,672460361
	LOC101889275	esterase B1-like	-0,32170	4,714909099	0,394	0,587469413
	LOC101890018	esterase FE4-like	0,37124	2,889402769	0,569	0,727740094
	LOC101889364	esterase-5A-like	2,09448	6,332141317	0,000	0,001381995
UGT	LOC101888811	UDP-glucuronosyltransferase 2A2-like	0,11829	3,385124693	0,841	0,913028031
	LOC101893116	UDP-glucuronosyltransferase 2A3-like	4,57714	4,899949527	0,000	2,8299E-12
	LOC101893291	UDP-glucuronosyltransferase 2A3-like	0,94181	6,540181742	0,040	0,185230118
	LOC101889193	UDP-glucuronosyltransferase 2A3-like	2,32228	4,256032423	0,000	0,0014777
	LOC101889322	UDP-glucuronosyltransferase 2B13-like	1,55901	6,01009183	0,001	0,012827254
	LOC101890271	UDP-glucuronosyltransferase 2B13-like	1,34061	4,612746069	0,005	0,054169611
	LOC101900184	UDP-glucuronosyltransferase 2B13-like	0,34764	4,881741127	0,384	0,578601573
	LOC101895816	UDP-glucuronosyltransferase 2B15-like	1,21846	5,716331269	0,017	0,116081042
	LOC101897252	UDP-glucuronosyltransferase 2B15-like	0,40370	1,532907397	0,489	0,665876962
	LOC101899999	UDP-glucuronosyltransferase 2B17-like	0,68725	7,542089237	0,069	0,242334501
	LOC101899504	UDP-glucuronosyltransferase 2B17-like	0,30430	4,745432103	0,466	0,648120567
	LOC101897074	UDP-glucuronosyltransferase 2B1-like	-0,01717	3,532212992	0,979	0,991920527
	LOC101893458	UDP-glucuronosyltransferase 2B1-like	-1,32340	4,818435119	0,072	0,247995578
	LOC101890612	UDP-glucuronosyltransferase 2B20-like	0,77593	3,86960014	0,148	0,345416637
	LOC101890444	UDP-glucuronosyltransferase 2B20-like	0,26086	4,493935334	0,604	0,756121556
	LOC101892660	UDP-glucuronosyltransferase 2B31-like	2,28767	5,727941067	0,000	3,17602E-05
	LOC101890269	UDP-glucuronosyltransferase 2B33-like	1,83572	3,412502334	0,001	0,012353676
	LOC101890707	UDP-glucuronosyltransferase 2B4-like	-2,37046	0,161243907	0,002	0,03059195
	LOC101889773	UDP-glucuronosyltransferase 2B7-like	2,34135	4,382231096	0,000	0,000554607
	LOC101893619	UDP-glucuronosyltransferase 2B9-like	-0,33415	0,967780928	0,662	0,797598898
	LOC101889496	UDP-glucuronosyltransferase 2C1-like	2,74065	3,61982905	0,000	7,79606E-05
	LOC101889147	UDP-glucuronosyltransferase-like	5,36461	2,905539956	0,000	1,82251E-10
	LOC101892938	UDP-glucuronosyltransferase-like	2,48309	3,405771085	0,000	0,000632598
	LOC101899032	UDP-glucuronosyltransferase-like	1,19735	2,971496127	0,028	0,151368553
	LOC101899202	UDP-glucuronosyltransferase-like	0,96948	2,372905438	0,090	0,275428198
	LOC101892765	UDP-glucuronosyltransferase-like	-0,27975	-0,03180039	0,711	0,828900035
	LOC101893798	UDP-glucuronosyltransferase-like	-0,41415	5,254465421	0,236	0,442570727

ABC	LOC101895261	ABC transporter F family member 4-like	-0,48667	5,354639716	0,223	0,432212813
	LOC101892798	ABC transporter G family member 1-like	0,19028	2,911271799	0,720	0,835574014
	LOC101895448	ABC transporter G family member 20-like	1,41023	4,041618078	0,036	0,17539141
	LOC101898054	ABC transporter G family member 20-like	1,33032	5,485663165	0,004	0,04124907
	LOC101889472	ABC transporter G family member 20-like	1,19678	5,394445632	0,028	0,151368553
	LOC101890903	ABC transporter G family member 22-like	-0,70937	6,031402911	0,079	0,261510465
	LOC101899230	ATP-binding cassette sub-family A member 13-like	-0,53020	5,729211924	0,109	0,299621827
	LOC101888352	ATP-binding cassette sub-family A member 3-like	0,42627	6,842363526	0,369	0,566194719
	LOC101895192	ATP-binding cassette sub-family B member 10 mitochondrial-lik	-0,43139	5,335480901	0,222	0,430024788
	LOC101899501	ATP-binding cassette sub-family B member 6 mitochondrial-like	0,26693	6,22456283	0,415	0,605411572
	LOC101888322	ATP-binding cassette sub-family B member 7 mitochondrial-like	0,08102	5,7493653	0,793	0,884040778
	LOC101888861	ATP-binding cassette sub-family B member 7 mitochondrial-like	-0,89166	2,54679236	0,082	0,264914143
	LOC101891270	ATP-binding cassette sub-family B member 8 mitochondrial-like	0,01729	5,949297353	0,956	0,979907424
	LOC101896101	ATP-binding cassette sub-family C member Sur-like	0,51878	2,524809115	0,373	0,568964314
	LOC101887509	ATP-binding cassette sub-family D member 2-like	0,15082	5,75693345	0,675	0,805029899
	LOC101901316	ATP-binding cassette sub-family D member 3-like	0,33950	6,932821961	0,370	0,567010414
	LOC101894209	ATP-binding cassette sub-family E member 1-like	-0,03998	8,793166779	0,913	0,956359941
	LOC101901102	ATP-binding cassette sub-family F member 1-like	-0,01606	7,435114771	0,965	0,985389777
	LOC101897819	ATP-binding cassette sub-family F member 2-like	0,25883	8,837344729	0,489	0,666241803
	LOC101901338	ATP-binding cassette sub-family F member 3-like	-0,05368	6,580129729	0,875	0,934557982
	LOC101894746	ATP-binding cassette sub-family G member 1-like	1,85306	2,834556957	0,006	0,059509333
	LOC101897551	ATP-binding cassette sub-family G member 1-like	1,79016	7,135374516	0,001	0,015273656
	LOC101894584	ATP-binding cassette sub-family G member 1-like	1,64231	5,540785046	0,003	0,035948018
	LOC101897386	ATP-binding cassette sub-family G member 1-like	1,39661	3,108324966	0,027	0,150468875
	LOC101897724	ATP-binding cassette sub-family G member 1-like	1,22259	4,187040751	0,044	0,192788431
	LOC101888695	ATP-binding cassette sub-family G member 1-like	0,58891	5,916190315	0,115	0,308808354
	LOC101896421	ATP-binding cassette sub-family G member 1-like	-0,22916	5,16961226	0,555	0,716404627
	LOC101890630	ATP-binding cassette sub-family G member 4-like	1,59163	2,213822518	0,017	0,113678191
	LOC101890462	ATP-binding cassette sub-family G member 4-like	1,56180	4,30821233	0,004	0,044681245
	LOC101897891	ATP-binding cassette sub-family G member 4-like	0,66301	0,897226221	0,400	0,593370618
	LOC101894909	ATP-binding cassette sub-family G member 4-like	1,32738	4,055415828	0,028	0,152339363
	LOC101887291	ATP-binding cassette sub-family G member 4-like	0,85416	5,797313455	0,015	0,107704717
	LOC101899158	ATP-binding cassette sub-family G member 5-like	0,85399	2,855070399	0,127	0,320858415
