# Supplementary material for: Analysis of Differentially Expressed Genes Related to Resistance in Spinosad- and Neonicotinoid-Resistant Musca domestica L. (Diptera: Muscidae) Strains
Source: PLoS One. 2017 Jan 26;12(1):e0170935. doi: 10.1371/journal.pone.0170935 (PMC5268453; doi:10.1371/journal.pone.0170935)
Supplement: S3 Table — Fold change (log2 values), logCPM, P-Value and FDR are provided. (PDF) [file pone.0170935.s003.pdf]

S3 Table. Raw data for global gene expression and expression of genes related to metabolism in the spinosad-resistant 791spin strain compared to the neonicotinoid-resistant 766b strain.

Fold change (log2 values), logCPM, P-Value and FDR are provided.

Gene	#feature	description	logFC	logCPM	PValue	FDR
Global expression	LOC101889469	cecropin-C-like	9,464703458	1,20357621	0,0000	0,000209521
	LOC101889899	U3 small nucleolar RNA-associated protein 6 homolog	8,768278774	0,71370225	0,0000	9,35542E-10
	LOC101890701	uncharacterized	8,604274676	3,79972186	0,0000	4,70855E-13
	LOC101899362	prolyl 4-hydroxylase subunit alpha-2-like	8,434953626	2,58511433	0,0000	4,92197E-18
	LOC101896904	uncharacterized	8,285126482	4,56235511	0,0000	1,34819E-15
	LOC101891140	uncharacterized	8,154748222	-0,5359986	0,0000	2,56857E-06
	LOC101889226	lysozyme 1-like	8,150192234	2,56002643	0,0000	1,56697E-08
	LOC101901700	putative nuclease HARBI1-like	8,130771574	1,51196131	0,0000	6,80725E-13
	LOC101899453	uncharacterized	7,956239821	0,79669778	0,0000	1,56697E-08
	LOC101887846	uncharacterized	7,932383575	2,91524835	0,0000	1,79055E-16
	LOC101899637	acyl-CoA-binding protein-like	7,661981069	3,19018334	0,0000	1,79055E-16
	LOC101896049	antichymotrypsin-2-like	7,12229092	2,70658868	0,0000	1,13505E-12
	LOC101888936	cecropin-A1-like	7,062407304	2,65368682	0,0000	0,000201586
	LOC101892852	uncharacterized	6,763975384	1,56967546	0,0000	3,39062E-09
	LOC101895611	aquaporin-like	6,71745131	3,28734578	0,0000	6,42034E-14
	LOC101899565	keratin-associated protein 19-2-like	6,449199133	4,26523607	0,0000	0,000272973
	LOC101895703	adult-specific cuticular protein ACP-20-like	6,294766487	-0,6519272	0,0000	1,11055E-05
	LOC101888410	lysozyme 1-like	6,255293697	3,79623052	0,0000	3,18505E-07
	LOC101888862	RNA-directed DNA polymerase from mobile element jockey-like	6,227422087	0,61951492	0,0000	1,52812E-07
	LOC101900010	Bardet-Biedl syndrome 4 protein homolog	6,220447033	1,69002117	0,0000	2,27494E-06
	LOC101888938	uncharacterized	6,164728949	1,7411261	0,0000	1,56697E-08
P450	LOC101900728	cytochrome P450 18a1-like	-0,900863353	4,00881839	0,0525	0,370326813
	LOC101888518	cytochrome P450 302a1 mitochondrial-like	-0,031598278	4,82102235	0,9333	1
	LOC101901255	cytochrome P450 306a1-like	0,214796849	1,81124864	0,6908	0,980996503
	LOC101900639	cytochrome P450 307a1-like	0,552844708	3,96219061	0,2567	0,79402241
	LOC101901025	cytochrome P450 315a1 mitochondrial-like	-1,17028217	1,92413381	0,0758	0,458127192
	LOC101898177	cytochrome P450 4ae1-like	-0,219892273	3,63533388	0,7036	0,984569892
	LOC101898930	cytochrome P450 4d1-like	-1,43769186	7,77031188	0,0289	0,267385566
	LOC101898004	cytochrome P450 4d2-like	0,61545205	0,6617891	0,3273	0,854893663
	LOC101897669	cytochrome P450 4d2-like	0,440996239	2,66409557	0,3871	0,893519781
	LOC101897841	cytochrome P450 4d2-like (4d56)	2,682186323	5,11336304	0,0000	2,65907E-05
	LOC101894425	cytochrome P450 4d8-like	0,4019277	-0,2872166	0,6190	0,96919129

LOC101892899	cytochrome P450 4d8-like	0,292928722	1,07281283	0,7111	0,985379706
LOC101896081	cytochrome P450 4e2-like	1,081378672	2,01598616	0,0976	0,517357648
LOC101895915	cytochrome P450 4e3-like	-1,128361017	5,25555101	0,0592	0,396796499
LOC101890640	cytochrome P450 4g15-like	-0,21540466	2,16149684	0,7533	0,98909752
LOC101887550	cytochrome P450 4g1-like (4g13)	-2,67713797	7,51975465	0,0068	0,108861322
LOC101887882	cytochrome P450 4g1-like (4g2)	-1,178031906	10,9320273	0,0823	0,477250022
LOC101889105	cytochrome P450 4g1-like (4g98)	-3,012613781	5,68957218	0,0001	0,003762045
LOC101891224	cytochrome P450 4p1-like	-1,011057848	4,87279934	0,1205	0,577191782
LOC101892246	cytochrome P450 6A1-like	-0,923879389	8,70704848	0,0905	0,500818282
LOC101892072	cytochrome P450 6A1-like	-1,379616808	6,70058832	0,0044	0,08198677
LOC101889365	cytochrome P450 6A1-like	0,843621967	1,11679531	0,2264	0,762398704
LOC101899899	cytochrome P450 6a2-like	1,741526617	4,35540392	0,0056	0,095408568
LOC101892970	cytochrome P450 6a8-like	-0,524986746	3,50386916	0,3646	0,879402055
LOC101894510	cytochrome P450 6a8-like	-0,722701748	1,24762945	0,4064	0,901215972
LOC101890041	cytochrome P450 6a9-like	1,266657862	4,1152196	0,0152	0,182024313
LOC101891761	cytochrome P450 6a9-like	1,025520807	3,35265895	0,0506	0,364574177
LOC101893114	cytochrome P450 6a9-like	0,678942833	2,29506613	0,2393	0,775732712
LOC101890373	cytochrome P450 6a9-like (6a37)	1,072763433	6,14688754	0,0099	0,137638119
LOC101900791	cytochrome P450 6d1-like	1,107605316	8,60546861	0,0150	0,180235993
LOC101899746	cytochrome P450 6d1-like	0,60282657	7,06555837	0,2725	0,807053665
LOC101889532	cytochrome P450 6d1-like	0,444368142	3,12730473	0,3579	0,874546655
LOC101899135	cytochrome P450 6d1-like	0,412647454	1,55729422	0,5207	0,940997081
LOC101900431	cytochrome P450 6d3-like	0,675002783	1,65479905	0,2760	0,810382325
LOC101899585	cytochrome P450 6d3-like	-1,06707544	7,00053588	0,0544	0,378408079
LOC101889269	cytochrome P450 6g1-like	0,08318039	-0,0164648	0,9101	1
LOC101898562	cytochrome P450 6g1-like (6g4)	-1,04758335	8,61436856	0,0419	0,33212761
LOC101890758	cytochrome P450 CYP12A2-like	1,899013461	1,59376373	0,0123	0,15861605
LOC101889672	cytochrome P450 CYP12A2-like	0,921140004	0,8640387	0,1934	0,720032657
LOC101889684	cytochrome P450 CYP12A2-like	0,569652213	6,21249754	0,1867	0,710317993
LOC101890931	cytochrome P450 CYP12A2-like	0,270592275	3,16375457	0,6297	0,971477985
LOC101891274	cytochrome P450 CYP12A2-like	-0,147803482	1,93290533	0,8334	0,996056133
LOC101889857	cytochrome P450 CYP12A2-like	-0,51067448	4,46822544	0,3667	0,880509326
LOC101896195	cytochrome P450 CYP12A2-like	-0,911278664	3,84011887	0,0793	0,469374176

LOC101898453	cytochrome P450 CYP12A2-like (12a1)	2,434928196	3,52043511	0,0003	0,011188897
LOC101889524	cytochrome P450 CYP12A2-like (12a14)	2,76910978	5,13175605	0,0000	1,99405E-05
LOC101893522	probable cytochrome P450 12c1 mitochondrial-like	-0,447414138	7,52746574	0,2296	0,76497835
LOC101892495	probable cytochrome P450 12c1 mitochondrial-like	0,959719443	7,01933537	0,0366	0,30945435
LOC101900938	probable cytochrome P450 28a5-like	0,010038208	2,15419462	0,9893	1
LOC101890714	probable cytochrome P450 28a5-like	1,089289031	5,69968282	0,0349	0,301865628
LOC101897279	probable cytochrome P450 28d1-like	-0,156879924	6,71897433	0,7566	0,989992246
LOC101891587	probable cytochrome P450 28d1-like	0,300307786	4,16278935	0,5910	0,964110617
LOC101897848	probable cytochrome P450 28d1-like	1,369051735	3,47461411	0,0073	0,113719244
LOC101893000	probable cytochrome P450 301a1 mitochondrial-like	-0,417945939	2,61673641	0,4605	0,920357857
LOC101889924	probable cytochrome P450 304a1-like	1,528723858	3,19848499	0,0266	0,256182845
LOC101895933	probable cytochrome P450 304a1-like	1,542928961	3,27688558	0,0228	0,236741994
LOC101890089	probable cytochrome P450 304a1-like	2,203631991	5,01743087	0,0001	0,005336327
LOC101898997	probable cytochrome P450 305a1-like	0,871598886	2,99580042	0,0968	0,516741585
LOC101900906	probable cytochrome P450 308a1-like	-0,303725384	7,42925451	0,5872	0,962270266
LOC101890335	probable cytochrome P450 309a2-like	-1,49541203	3,88323314	0,0421	0,332266822
LOC101899919	probable cytochrome P450 310a1-like	-1,184176711	0,49995593	0,0806	0,474354087
LOC101887394	probable cytochrome P450 311a1-like	-1,10052826	3,27454239	0,0746	0,455467007
LOC101896650	probable cytochrome P450 313a4-like	-0,135523828	2,86685018	0,8289	0,995860679
LOC101893162	probable cytochrome P450 313a4-like	-0,354491838	3,56858041	0,5279	0,94455337
LOC101896297	probable cytochrome P450 313a4-like	1,079956057	0,76042859	0,2937	0,825391604
LOC101890728	probable cytochrome P450 313a4-like	1,713799558	0,87229753	0,0828	0,478394153
LOC101896469	probable cytochrome P450 313a4-like	1,218893786	6,05532499	0,0577	0,392151964
LOC101891061	probable cytochrome P450 317a1-like	0,420391352	3,57641226	0,4589	0,920357857
LOC101892636	probable cytochrome P450 318a1-like	0,274019245	2,67783539	0,6578	0,976365253
LOC101887655	probable cytochrome P450 49a1-like	0,488088748	1,33707395	0,4558	0,919728028
LOC101901643	probable cytochrome P450 4ac1-like	-1,222749682	4,18476301	0,0869	0,489899065
LOC101897760	probable cytochrome P450 4ad1-like	1,668129746	3,67830178	0,0021	0,049023717
LOC101897033	probable cytochrome P450 4d14-like	0,074900049	0,584512	0,9137	1
LOC101891759	probable cytochrome P450 4d14-like	-0,926209079	6,25372695	0,0612	0,404543202
LOC101897209	probable cytochrome P450 4d14-like (4d9)	3,058443136	3,9630492	0,0000	0,000594735
LOC101891931	probable cytochrome P450 4d14-like	0,403964362	3,8248931	0,4414	0,914394316
LOC101893472	probable cytochrome P450 4p3-like	1,056806823	4,1562985	0,0585	0,395124463

	LOC101892717	probable cytochrome P450 4s3-like	-0,37400182	3,37234408	0,4413	0,91428802
	LOC101891157	probable cytochrome P450 4s3-like	0,866549207	4,61489609	0,0515	0,367542327
	LOC101895233	probable cytochrome P450 4aa1-like	-0,930406594	2,297214	0,2207	0,755249968
	LOC101891933	probable cytochrome P450 6a13-like	1,426530159	3,88520925	0,0063	0,103917056
	LOC101890715	probable cytochrome P450 6a14-like	0,004246026	4,26099695	0,9947	1
	LOC101892622	probable cytochrome P450 6a14-like	-0,231079524	0,97184629	0,7407	0,988174666
	LOC101890889	probable cytochrome P450 6a14-like	-0,314000415	3,68396364	0,5600	0,956889751
	LOC101892108	probable cytochrome P450 6a14-like	1,198254273	0,21348044	0,1097	0,552593024
	LOC101892278	probable cytochrome P450 6a14-like	1,223758797	1,41208987	0,0451	0,342476249
	LOC101892447	probable cytochrome P450 6a14-like	1,493506423	1,42678969	0,0111	0,148217182
	LOC101900065	probable cytochrome P450 6a14-like	2,775386335	6,9399059	0,0000	0,001907911
	LOC101887482	probable cytochrome P450 6a17-like	0,52649103	1,02251202	0,3752	0,888097695
	LOC101890543	probable cytochrome P450 6a17-like	1,549867739	2,15787574	0,0254	0,251238058
	LOC101898668	probable cytochrome P450 6a18-like	1,160676768	4,55997957	0,0078	0,11866222
	LOC101895803	probable cytochrome P450 6a21-like	0,463658265	4,30498377	0,4133	0,904972637
	LOC101890199	probable cytochrome P450 6a21-like	0,865865787	4,37683248	0,0667	0,429037327
	LOC101892586	probable cytochrome P450 6a21-like	1,020917761	3,57867834	0,0667	0,429037327
	LOC101892931	probable cytochrome P450 6a21-like	1,402676327	4,22492629	0,0156	0,185247938
	LOC101889539	probable cytochrome P450 6a21-like (6a36)	1,530297895	3,00938428	0,0060	0,099926069
	LOC101891408	probable cytochrome P450 6a21-like (6a58)	2,003328639	1,82974445	0,0026	0,058152363
	LOC101889704	probable cytochrome P450 6a21-like (6a7)	2,899258359	1,26624901	0,0001	0,003476182
	LOC101891297	probable cytochrome P450 6d5-like (6d8)	-1,818988444	6,97879819	0,0275	0,261441485
	LOC101900444	probable cytochrome P450 6g2-like (6g7)	-3,288724918	-0,1899066	0,0010	0,02763703
	LOC101899434	probable cytochrome P450 6t3-like	-0,365618116	-0,2034601	0,6664	0,977561537
	LOC101887226	probable cytochrome P450 6u1-like	-0,130465131	4,96233625	0,7279	0,987401415
	LOC101896204	probable cytochrome P450 6v1-like	-0,347964608	4,81174336	0,3403	0,864538388
	LOC101898775	probable cytochrome P450 9f2-like	-0,532964855	3,21551247	0,3096	0,84082269
	LOC101898942	probable cytochrome P450 9f2-like	-1,188192643	3,4214139	0,0339	0,296465602
	LOC101899118	probable cytochrome P450 9f2-like	-1,615976734	2,68023495	0,0125	0,160127313
	LOC101900658	probable cytochrome P450 9f2-like	1,667162287	4,31191641	0,0046	0,084381511
	LOC101898478	probable cytochrome P450 9f2-like	-0,332310607	7,6416413	0,5103	0,937019842
GST	LOC101897621	glutathione S-transferase 1-1-like	4,275948424	5,54476569	0,0000	6,80725E-13
	LOC101895956	glutathione S-transferase 1-1-like	0,661505375	4,55246057	0,1327	0,60945874

	LOC101887423	glutathione S-transferase 1-like	2,078494796	8,53882964	0,0000	0,00212253
	LOC101895555	glutathione S-transferase 1-like	2,053246675	8,15282183	0,0001	0,004476061
	LOC101887250	glutathione S-transferase 1-like	1,855957436	7,10011928	0,0005	0,018361747
	LOC101888181	glutathione S-transferase 1-like	1,473304131	1,03835595	0,0542	0,378243282
	LOC101895036	glutathione S-transferase 1-like	1,24351637	6,46570106	0,0061	0,101244093
	LOC101888349	glutathione S-transferase 1-like	0,949958327	1,40957621	0,1186	0,573357892
	LOC101899848	glutathione S-transferase 1-like	0,840489003	3,95498324	0,0999	0,525866928
	LOC101900672	glutathione S-transferase 1-like	0,804344245	7,79500807	0,0515	0,367542327
	LOC101895607	glutathione S-transferase 1-like	0,513201984	5,23259697	0,1561	0,658522593
	LOC101900016	glutathione S-transferase 1-like	0,337967725	3,88306135	0,4937	0,930203989
	LOC101894873	glutathione S-transferase 1-like	-0,361753892	7,45451	0,4682	0,92375588
	LOC101895316	glutathione S-transferase 1-like	-0,394718004	7,57525015	0,3262	0,853434382
	LOC101897277	glutathione S-transferase 2-like	1,338681072	5,47917824	0,0018	0,043219339
	LOC101897094	glutathione S-transferase 2-like	-0,484564262	4,67960407	0,2465	0,783382786
	LOC101897797	glutathione S-transferase D7-like	1,483984809	0,35735355	0,0504	0,364132039
	LOC101891696	glutathione S-transferase omega-1-like	0,152448866	6,26066066	0,6341	0,971858356
	LOC101888110	glutathione S-transferase theta-1-like	2,047399679	2,29525951	0,0010	0,027638311
	LOC101898455	glutathione S-transferase theta-1-like	0,970776043	2,58639472	0,0793	0,469374176
	LOC101890402	glutathione S-transferase theta-1-like	0,738243988	2,35099438	0,2332	0,769490812
	LOC101900949	glutathione S-transferase theta-1-like	0,612699032	5,11414214	0,0837	0,479400164
	LOC101897781	glutathione S-transferase theta-1-like	0,054328446	5,28232924	0,9041	1
	LOC101890455	glutathione S-transferase-like	0,007327559	6,61667298	0,9881	1
Esterase	LOC101896445	esterase B1-like	0,918812949	3,90629211	0,0643	0,419044255
	LOC101896978	esterase B1-like	0,490385486	7,21230271	0,2791	0,81363294
	LOC101896807	esterase B1-like	0,432065439	6,5979983	0,2914	0,822886968
	LOC101900490	esterase B1-like	0,319760971	3,93479334	0,5570	0,956562791
	LOC101897501	esterase B1-like	0,185019529	5,61158515	0,5922	0,964110617
	LOC101889275	esterase B1-like	0,071111408	4,7149091	0,8531	0,998980589
	LOC101896625	esterase B1-like	-0,407484381	8,41730936	0,4730	0,925373257
	LOC101897334	esterase B1-like	-0,581084322	4,68349343	0,3472	0,868840856
	LOC101898347	esterase B1-like	-0,636103384	4,49479826	0,2599	0,798620909
	LOC101897154	esterase B1-like	-0,766531833	5,74301209	0,1032	0,535897494
	LOC101895121	esterase B1-like	-0,796202523	3,21185692	0,1996	0,726748751

	LOC101898526	esterase B1-like	-0,972648956	3,85857058	0,1584	0,664538378
	LOC101898354	esterase B1-like	-1,012058758	1,43777493	0,1143	0,564475968
	LOC101898698	esterase B1-like	-2,357939989	2,9763668	0,0069	0,109489787
	LOC101890018	esterase FE4-like	1,494307674	2,88940277	0,0234	0,238814396
	LOC101889364	esterase-5A-like	-1,007392655	6,33214132	0,0394	0,32027413
UGT	LOC101888811	UDP-glucuronosyltransferase 2A2-like	1,605973903	3,38512469	0,0069	0,109489787
	LOC101893291	UDP-glucuronosyltransferase 2A3-like	1,23296233	6,54018174	0,0075	0,115839682
	LOC101893116	UDP-glucuronosyltransferase 2A3-like	-1,2373838	4,89994953	0,0149	0,179271882
	LOC101889193	UDP-glucuronosyltransferase 2A3-like	-0,090674088	4,25603242	0,8671	1
	LOC101889322	UDP-glucuronosyltransferase 2B13-like	1,508707619	6,01009183	0,0009	0,026517106
	LOC101900184	UDP-glucuronosyltransferase 2B13-like	0,837293197	4,88174113	0,0362	0,307599376
	LOC101890271	UDP-glucuronosyltransferase 2B13-like	0,82143301	4,61274607	0,0822	0,477250022
	LOC101897252	UDP-glucuronosyltransferase 2B15-like	1,034617997	1,5329074	0,0697	0,440046924
	LOC101895816	UDP-glucuronosyltransferase 2B15-like	0,809318057	5,71633127	0,1111	0,55746126
	LOC101899504	UDP-glucuronosyltransferase 2B17-like	0,160835904	4,7454321	0,6999	0,98398565
	LOC101899999	UDP-glucuronosyltransferase 2B17-like	0,1086721	7,54208924	0,7726	0,991754338
	LOC101893458	UDP-glucuronosyltransferase 2B1-like	3,526965217	4,81843512	0,0000	0,000659838
	LOC101897074	UDP-glucuronosyltransferase 2B1-like	0,940233179	3,53221299	0,1344	0,614893387
	LOC101890444	UDP-glucuronosyltransferase 2B20-like	0,780597611	4,49393533	0,1209	0,57763695
	LOC101890612	UDP-glucuronosyltransferase 2B20-like	-0,123621572	3,86960014	0,8207	0,994449487
	LOC101892660	UDP-glucuronosyltransferase 2B31-like	-1,270822377	5,72794107	0,0035	0,070857852
	LOC101890269	UDP-glucuronosyltransferase 2B33-like	-0,388136055	3,41250233	0,4569	0,919728028
	LOC101890707	UDP-glucuronosyltransferase 2B4-like	1,901513155	0,16124391	0,0173	0,197681202
	LOC101889773	UDP-glucuronosyltransferase 2B7-like	-0,32548724	4,3822311	0,5203	0,940997081
	LOC101893619	UDP-glucuronosyltransferase 2B9-like	-0,327023548	0,96778093	0,6888	0,980696151
	LOC101889496	UDP-glucuronosyltransferase 2C1-like	-1,209593219	3,61982905	0,0233	0,238541401
	LOC101889147	UDP-glucuronosyltransferase-like	0,244806826	2,90553996	0,6762	0,979418291
	LOC101893798	UDP-glucuronosyltransferase-like	0,004546546	5,25446542	0,9920	1
	LOC101892765	UDP-glucuronosyltransferase-like	-2,236820347	-0,0318004	0,0069	0,109113893
	LOC101899202	UDP-glucuronosyltransferase-like	-2,588143214	2,37290544	0,0000	0,001526894
	LOC101899032	UDP-glucuronosyltransferase-like	-3,702551866	2,97149613	0,0000	3,2106E-07
	LOC101892938	UDP-glucuronosyltransferase-like	-3,888775205	3,40577109	0,0000	5,61968E-08
ABC	LOC101895261	ABC transporter F family member 4-like	0,048263076	5,35463972	0,9059	1

LOC101892798	ABC transporter G family member 1-like	-0,308420145	2,9112718	0,5654	0,957281165
LOC101895448	ABC transporter G family member 20-like	0,036377538	4,04161808	0,9565	1
LOC101889472	ABC transporter G family member 20-like	0,540391029	5,39444563	0,3149	0,845069632
LOC101898054	ABC transporter G family member 20-like	-0,452709258	5,48566316	0,3148	0,845069632
LOC101890903	ABC transporter G family member 22-like	-0,134698879	6,03140291	0,7376	0,988174666
LOC101899230	ATP-binding cassette sub-family A member 13-like	0,243659477	5,72921192	0,4620	0,920357857
LOC101888352	ATP-binding cassette sub-family A member 3-like	0,155138601	6,84236353	0,7433	0,988366728
LOC101895192	ATP-binding cassette sub-family B member 10 mitochondrial-like	-0,093852762	5,3354809	0,7908	0,992959043
LOC101899501	ATP-binding cassette sub-family B member 6 mitochondrial-like	-0,757185763	6,22456283	0,0217	0,231293176
LOC101888861	ATP-binding cassette sub-family B member 7 mitochondrial-like	0,189687019	2,54679236	0,7101	0,985379706
LOC101888322	ATP-binding cassette sub-family B member 7 mitochondrial-like	-0,169237583	5,7493653	0,5849	0,96090874
LOC101891270	ATP-binding cassette sub-family B member 8 mitochondrial-like	0,04939713	5,94929735	0,8721	1
LOC101896101	ATP-binding cassette sub-family C member Sur-like	-1,039076791	2,52480912	0,0784	0,467841279
LOC101887509	ATP-binding cassette sub-family D member 2-like	-0,027154677	5,75693345	0,9419	1
LOC101901316	ATP-binding cassette sub-family D member 3-like	-0,011638987	6,93282196	0,9760	1
LOC101894209	ATP-binding cassette sub-family E member 1-like	-0,511905839	8,79316678	0,1630	0,671427099
LOC101901102	ATP-binding cassette sub-family F member 1-like	0,203049461	7,43511477	0,5781	0,958553857
LOC101897819	ATP-binding cassette sub-family F member 2-like	-0,571845337	8,83734473	0,1276	0,596955592
LOC101901338	ATP-binding cassette sub-family F member 3-like	-0,320229421	6,58012973	0,3467	0,868840856
LOC101897386	ATP-binding cassette sub-family G member 1-like	0,149783497	3,10832497	0,8076	0,992959043
LOC101894746	ATP-binding cassette sub-family G member 1-like	0,113151946	2,83455696	0,8637	1
LOC101894584	ATP-binding cassette sub-family G member 1-like	-0,406422691	5,54078505	0,4505	0,918443871
LOC101896421	ATP-binding cassette sub-family G member 1-like	-0,727125785	5,16961226	0,0622	0,408474007
LOC101888695	ATP-binding cassette sub-family G member 1-like	-0,729236802	5,91619031	0,0520	0,368278538
LOC101897724	ATP-binding cassette sub-family G member 1-like	-0,750794198	4,18704075	0,2126	0,748357163
LOC101897551	ATP-binding cassette sub-family G member 1-like	-0,820003059	7,13537452	0,1176	0,571328908
LOC101897891	ATP-binding cassette sub-family G member 4-like	0,659844354	0,89722622	0,3849	0,891482567
LOC101890630	ATP-binding cassette sub-family G member 4-like	0,588623847	2,21382252	0,3612	0,877042304
LOC101890462	ATP-binding cassette sub-family G member 4-like	-0,396370914	4,30821233	0,4574	0,919728028
LOC101887291	ATP-binding cassette sub-family G member 4-like	-0,059185762	5,79731345	0,8672	1
LOC101894909	ATP-binding cassette sub-family G member 4-like	-0,418650464	4,05541583	0,4848	0,927846887
LOC101899158	ATP-binding cassette sub-family G member 5-like	0,165254209	2,8550704	0,7632	0,99085096
